# Activation of STAT3 integrates common profibrotic pathways to promote fibroblast activation and tissue fibrosis

**DOI:** 10.1038/s41467-017-01236-6

**Published:** 2017-10-24

**Authors:** Debomita Chakraborty, Barbora Šumová, Tatjana Mallano, Chih-Wei Chen, Alfiya Distler, Christina Bergmann, Ingo Ludolph, Raymund E. Horch, Kolja Gelse, Andreas Ramming, Oliver Distler, Georg Schett, Ladislav Šenolt, Jörg H. W. Distler

**Affiliations:** 1 Department of Internal Medicine 3 – Rheumatology and Immunology, Friedrich-Alexander-University Erlangen-Nürnberg (FAU) and University Hospital Erlangen, Erlangen, 91054 Germany; 2 Institute of Rheumatology and Department of Rheumatology, First Faculty of Medicine, Charles University, Prague, 120 00 Czech Republic; 3 Department of Plastic and Hand Surgery and Laboratory for Tissue Engineering and Regenerative Medicine, University Hospital of Erlangen, Friedrich-Alexander University of Erlangen-Nürnberg (FAU), Erlangen, 91054 Germany; 4 Department of Orthopaedic Trauma Surgery, University Hospital Erlangen, Friedrich-Alexander University of Erlangen-Nürnberg (FAU), Erlangen, 91054 Germany; 5 Center of Experimental Rheumatology and Zurich Center of Integrative Human Physiology, University Hospital Zurich, Zurich, 8091 Switzerland

**Keywords:** Systemic sclerosis, Transforming growth factor beta

## Abstract

Signal transducer and activator of transcription 3 (STAT3) is phosphorylated by various kinases, several of which have been implicated in aberrant fibroblast activation in fibrotic diseases including systemic sclerosis (SSc). Here we show that profibrotic signals converge on STAT3 and that STAT3 may be an important molecular checkpoint for tissue fibrosis. STAT3 signaling is hyperactivated in SSc in a TGFβ-dependent manner. Expression profiling and functional studies in vitro and in vivo demonstrate that STAT3 activation is mediated by the combined action of JAK, SRC, c-ABL, and JNK kinases. STAT3-deficient fibroblasts are less sensitive to the pro-fibrotic effects of TGFβ. Fibroblast-specific knockout of STAT3, or its pharmacological inhibition, ameliorate skin fibrosis in experimental mouse models. STAT3 thus integrates several profibrotic signals and might be a core mediator of fibrosis. Considering that several STAT3 inhibitors are currently tested in clinical trials, STAT3 might be a candidate for molecular targeted therapies of SSc.

## Abstract (web summary)

STAT3 is a transcription factor that is activated in fibrotic diseases such as systemic sclerosis. Here the authors show that STAT3 is the converging point for multiple pro-fibrotic signalling pathways, and that its genetic ablation or inhibition ameliorate skin fibrosis in mouse models.

## Introduction

Fibrotic diseases impose a major socioeconomic burden on modern societies and have been estimated to account for 45% of deaths in the developed world^1^. Their common histopathological feature is an excessive accumulation of extracellular matrix, which disrupts the physiological tissue architecture^2^. Tissue fibrosis can occur after defined stimuli with a subsequent inflammatory response, but in many fibrotic diseases, no initiating trigger can be identified. These idiopathic fibrotic diseases can manifest on virtually every organ. A prototypical systemic idiopathic fibrotic disease is systemic sclerosis (SSc)^3^. Failure of the affected organs is common in SSc and results in high morbidity and mortality and no targeted therapies are yet available for the treatment of fibrosis in SSc^2,3^. Myofibroblasts are the principle source of extracellular matrix during physiologic tissue repair as well as in fibrotic diseases. However, while tissue remodeling is tightly controlled in normal wound healing and is turned off as soon as the damage is repaired, the fibroblasts escape this regulation^1^. Myofibroblast differentiation may initially be depended on profibrotic cytokines, but with prolonged external stimulation, myofibroblasts become independent of external stimuli and remain persistently activated in fibrotic diseases.

Although the molecular mechanisms leading to aberrant activation of fibroblasts are incompletely understood, transforming growth factor-β (TGFβ) has been identified as a core pathway of fibrosis^2–5^. The levels of TGFβ are increased in fibrotic diseases and fibroblasts display activation of TGFβ signaling with increased transcription of TGFβ target genes^4,6^. Moreover, stimulation of resting fibroblasts with TGFβ induces an activated myofibroblast phenotype and a gene expression profile in resting fibroblasts that is reminiscent of SSc fibroblasts^6,7^. The key role of TGFβ signaling in the pathogenesis of fibrosis is further demonstrated by the development of systemic fibrosis in mice with fibroblast-specific overexpression of constitutively active TGFβ receptor type I (TBRact)^8^. The effects of TGFβ are mediated by a complex network of intracellular signaling events. SMAD proteins that mediate canonical TGFβ signaling are considered as major intracellular mediators^9^. SMAD independent, so-called non-canonical TGFβ pathways, such as mitogen activated involving the mitogen-activated protein kinases (MAPKs), focal adhesion kinase (FAK), the tyrosine kinase c-ABL, and early growth response 1 are also transducing in the pro-fibrotic effects of TGFβ^10,11^. However, inhibition of these downstream pathways does not completely abrogate the pro-fibrotic effects of TGFβ^12–14^, indicating that additional pathways are important to transduce the stimulatory effects of TGFβ. Identification of these novel downstream mediators of TGFβ might have translational implications and may provide the basis for novel-targeted therapies for fibrotic diseases.

Signal transducer and activator of transcription 3 (STAT3) was originally identified as an interleukin-6-activated transcription factor and subsequently reported to also transduce signals from several other stimuli including additional cytokines, hormones, and growth factors^15,16^. Upon binding of these ligands to their receptors, STAT3 is activated by phosphorylation at Tyr-705 in the STAT3 transactivation domain. Phosphorylation of STAT3 at Tyr-705 can be executed by kinases of the JAK and SRC families, but also by other receptor- and non-receptor-associated tyrosine kinases, such as JNK and c-ABL^16–19^. Phosphorylation at Tyr-705 is essentially required for STAT3 signaling as it allows STAT3 to dimerize, translocate to the nucleus, and to modulate the transcription of target genes and is thus commonly used to assess the activation of STAT3 signaling. STAT3 regulates fundamental cellular processes including inflammation, cell growth, proliferation, differentiation, migration, and apoptosis^20^. Given its broad regulatory effects, it may not be surprising that deregulation of STAT3 signaling has been linked to the pathogenesis of various diseases. STAT3 is an oncogenic transcription factor and constitutive activation of STAT3 has been observed in numerous malignancies^15,16,21–23^. STAT3 is activated by several pro-inflammatory cytokines, including interleukin-6 (IL-6)^24–26^, which is a prime target for therapeutic intervention in several inflammatory diseases including rheumatoid arthritis, Still’s disease and giant cell arthritis. The levels of IL-6 are also increased in SSc and treatment with monoclonal antibodies against IL-6 receptor may improve clinical outcomes in an inflammatory subgroup of SSc patients^27^. Of particular interest, STAT3 has also been linked to mesenchymal tissue responses during development and in cancer. STAT3 is essentially required for mesoderm induction in Xenopus embryos^28^. STAT3 has very recently been found to promote tumor progression by promoting desmoplastic reactions and tumor invasion^29,30^. The identification of STAT3 as a potential therapeutic target in various diseases promoted the development of STAT3 inhibitors and these efforts generated numerous candidates^22–24,31^. First of those inhibitors have already been tested in clinical trials with promising results.

Several of the upstream kinases that regulate phosphorylation and thus activation of STAT3, such as JAK2, JNK, and SRC, have previously been characterized as mediators of non-canonical TGFβ signaling^6,13,17,32^. Given the convergence of those mediators toward STAT3 activation, we hypothesized that STAT3, in addition to its central role in inflammation, may be a crucial checkpoint for fibroblast activation and tissue fibrosis.

In this study, we demonstrate that STAT3 signaling is hyperactive in SSc by the combined action of JAK, SRC, c-ABL, and JNK kinases. Pharmacological or genetic inactivation of STAT3 inhibits TGFβ-induced fibroblast-to-myofibroblast transition and collagen release in cultured fibroblasts and ameliorates skin fibrosis in two mouse models of SSc. We thus demonstrate that STAT3 is a central integrator of multiple profibrotic signals and a candidate for molecular-targeted therapies of fibrosis in SSc.

## Results

### Phosphorylated STAT3 accumulates in fibrotic skin

**Fig. 1:**
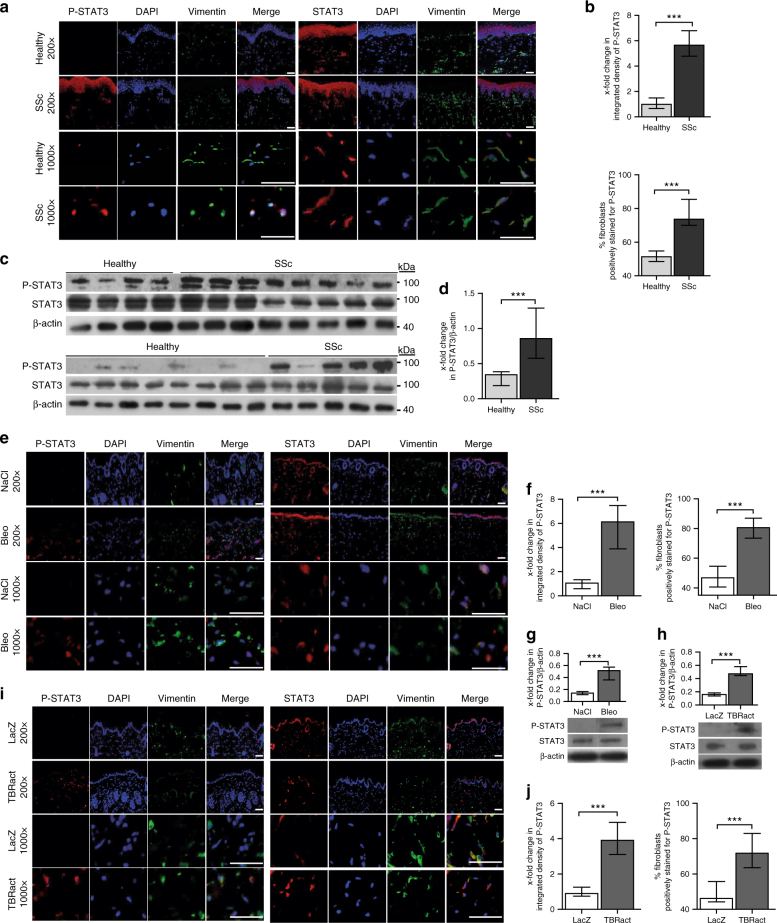
Activation of STAT3 signaling in fibrotic SSc skin. **a**–**d** Evaluation of STAT3 signaling in human samples: **a** Representative images and **b** quantitative analysis of immunofluorescence staining for P-STAT3 (left) and total STAT3 (right) co-stained with the vimentin (fibroblast marker) and DAPI (staining of nuclei) shown at 200-fold and 1000-fold magnification (*n* = 12 for SSc and *n* = 10 for healthy skin). **c** Western blots and **d** quantification of the levels of P-STAT3 and total STAT3 in human dermal fibroblasts from 13 SSc patients and 12 healthy individuals. **e**–**g** Evaluation of STAT3 signaling in the mouse model of bleomycin-induced skin fibrosis: **e** Representative images of immunofluorescence stainings (200-fold and 1000-fold magnification) showing P-STAT3 (left) and total STAT3 (right) along with **f** quantification and **g** confirmation by western blot analyses in the skin of mice injected with NaCl or bleomycin (*n* ≥ 6 for each group). **h**–**j** Evaluation of STAT3 signaling in the mouse model of TBRact-induced fibrosis: **h** Western blot and **i**, **j** immunofluorescence analyses of P-STAT3 expression in the skin of mice injected with TBRact. *N* ≥ 6 for each group with two or three technical replicates for all experiments. Expected band size for P-STAT3 and STAT3 are 79 kDa (lower faint band) and 86 kDa (higher intense band) and the ladder represents 100 kDa. Beta-actin expected molecular weight/size is 42 kDa. Horizontal scale bar, 100 μm. Results are shown as median ± interquartile range (IQR). Significance was determined by Mann–Whitney test, as compared to healthy volunteers or with non-fibrotic control mice, respectively. *P < 0.05; **P < 0.01, ***P < 0.001

We first analyze whether STAT3 signaling is activated by analyzing the levels of STAT3 phosphorylated at tyrosine 705 (P-STAT3), the common readout for STAT3 activation, in the skin of SSc patients as compared to age- and sex-matched healthy volunteers. P-STAT3 accumulates in the SSc skin with prominent staining in spindle-shaped cells in the dermis, whereas only few cells in the dermis stains positive for P-STAT3 in healthy individuals (Fig. 1a–d). Costaining of P-STAT3 with vimentin demonstrates that 84 ± 7% of SSc fibroblasts stain positive for P-STAT3, as compared to only 34 ± 3% in healthy skin (*P* < 0.0001 by Mann–Whitney *U*-test) (Fig. 1a, c). Consistent with the persistent activation of SSc fibroblasts under culture conditions, we observe an increased accumulation of P-STAT3 in SSc fibroblasts even after several passages in culture (Fig. 1b, d). The activation of STAT3 signaling in SSc is also mimicked in experimental models of skin fibrosis. Challenge of mice with bleomycin or overexpression of TBRact in the skin of mice significantly increases the levels of P-STAT3 as compared to non-fibrotic control mice (Fig. 1e–j).

### TGFβ induces phosphorylation of STAT3

**Fig. 2:**
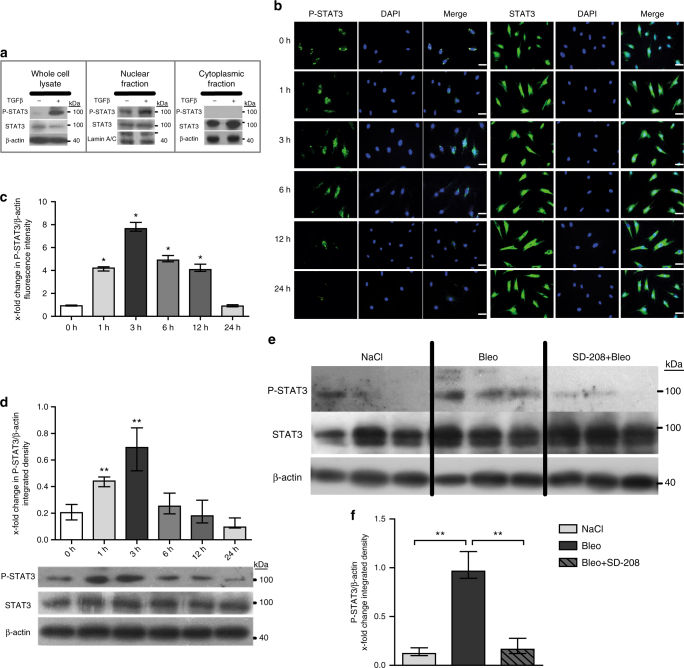
TGFβ activates STAT3 signaling in fibroblasts. **a** Total nuclear and cytoplasmatic levels of P-STAT3 and total STAT3 in human dermal fibroblasts stimulated for 1 h with TGFβ as analyzed by western blot (*n* ≥ 4 and 2 technical replicates). **b**–**d** Time-dependent changes in the levels of P-STAT3 as evaluated by **b** immunofluorescence staining and **c** quantification further confirmed by **d** western blot (*n* ≥ 5 for all). **e**, **f** Levels of P-STAT3 in fibroblasts in bleomycin-challenged mice, treated with SD-208, a selective TGFβ receptor type 1 kinase inhibitor (*n* ≥ 4 with 2 technical replicates for all groups) analyzed by **e** western blot and **f** its quantification. Representative blots and images (200-fold magnification; horizontal scale bar, 100 μm) are shown. Expected band size for P-STAT3 and STAT3 are 79 kDa (lower faint band) and 86 kDa (higher intense band) and the ladder represents 100 kDa. Beta-actin expected molecular weight/size is 42 kDa. Results are shown as median ± interquartile range (IQR). *P < 0.05, **P < 0.01 vs. healthy dermal fibroblasts or unstimulated/untreated control fibroblasts, or vehicle-treated, fibrotic mice, respectively

Given the consistent activation of STAT3 signaling in fibroblasts of SSc patients and in different experimental models, we speculate that a core pathway such as TGFβ might drive the activation. Indeed, recombinant TGFβ induces phosphorylation of STAT3 in cultured fibroblasts, with maximum accumulation of P-STAT3 observed after 3 h of TGFβ stimulation (Fig. 2a–d). Immunofluorescence staining further confirms the activation of STAT3 signaling by TGFβ and demonstrates nuclear localization of P-STAT3 in TGFβ-stimulated fibroblasts (Fig. 2b–c). Furthermore, selective inhibition of TGFβ signaling by SD-208, a TGFβ receptor I inhibitor^33^, prevents the upregulation of P-STAT3 in bleomycin-challenged mice (Fig. 2e–f). Taken together, these results demonstrate that P-STAT3 is overexpressed in SSc fibroblasts in a TGFβ-dependent manner.

### JNK JAK SRC and c-ABL kinases jointly activate STAT3 signaling in fibroblasts in response to TGFβ

**Fig. 3:**
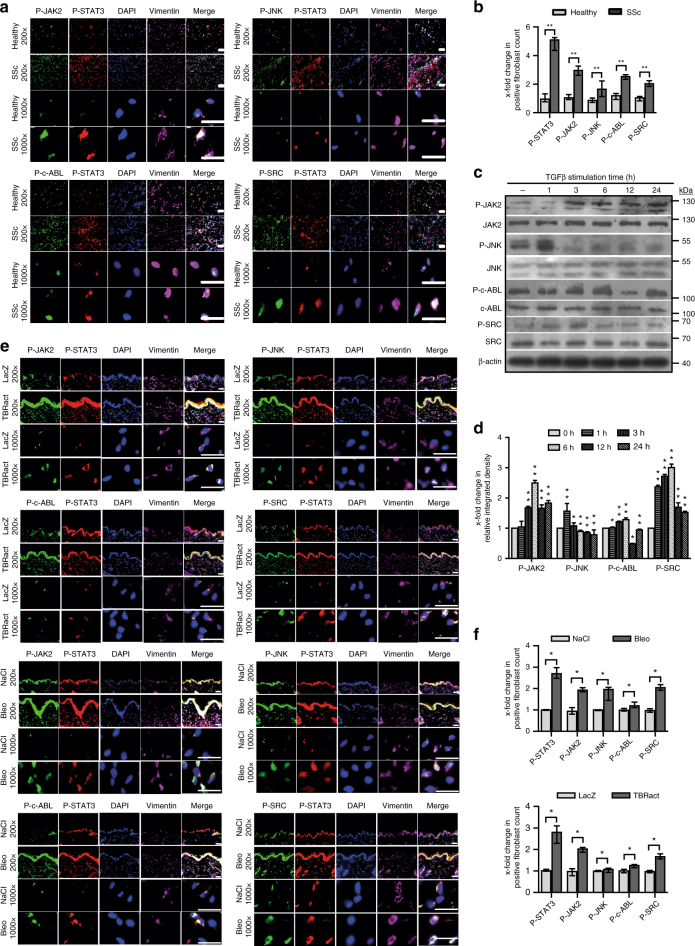
Activation of JAK2, JNK, SRC, and c-ABL in SSc. **a**, **b** Representative images (**a**) and quantification (**b**) of immunofluorescence stainings for P-SRC, P-JAK2, P-JNK, or P-c-ABL co-stained with P-STAT3, DAPI, and vimentin in skin sections of healthy individuals and SSc patients (*n* ≥ 4 with 3 technical replicates per group for all experiments). **c** Western blots and **d** quantification of the activation of SRC, JAK2, JNK, and c-ABL in human dermal fibroblasts stimulated with TGFβ for different time periods (*n* ≥ 4 and 2 technical replicates for all). A representative western blot and quantification of four independent experiments are shown. **e** Representative images and **f** quantification of immunofluorescence stainings for P-SRC, P-JNK, P- JAK2, and P-c-ABL along with P-STAT3 in the skin of mice challenged with bleomycin or overexpressing TBRact with respective non-fibrotic control mice (*n* ≥ 4 mice with 3 technical replicates per group for all experiments). Magnifications of 200-fold and 1000-fold are shown for all immunofluorescence stainings (horizontal scale bar, 100 μm). JAK2 (expected molecular size, 132 kDa), JNK (expected molecular size, 49 and 55 kDa), c-ABL (expected molecular size, 125–135 kDa), and SRC (expected molecular size, 60 kDa) are represented by ladders showing 130, 55, 100, and 70 kDa, respectively. Beta-actin expected molecular weight/size is 42 kDa. Results are shown as median ± interquartile range (IQR). Significance was determined by Mann–Whitney test, as compared to healthy individuals, unstimulated fibroblasts or with non-fibrotic control mice, respectively.*P < 0.05; **P < 0.01

We next evaluate which upstream kinases mediate phosphorylation of STAT3 in response to TGFβ. Immunofluorescence staining shows activation of JAK2, JNK, SRC, and c-ABL signaling in SSc with increased staining for P-JAK2 (Tyr-1007/1008), P-JNK (Thr-183/ Tyr-185), P-SRC (Tyr-416), and P-c-ABL (Tyr-412) in fibroblasts of SSc skin as compared to skin from healthy individuals (Fig. 3a, b). Stimulation of cultured human dermal fibroblasts with recombinant TGFβ also promotes accumulation of P-JAK2, P-JNK, P-SRC, and P-c-ABL in a time-dependent manner (Fig. 3c, d). We thus inhibit JAK2, SRC, JNK, and c-ABL, all of which have also been demonstrated to phosphorylate STAT3 and are known to transduce the pro-fibrotic effects of TGFβ from the cell surface to the nucleus^13,18,19,34,35^. P-JAK2, P-JNK, P-SRC, and P-c-ABL accumulate in fibroblasts in bleomycin- and TBRact-induced experimental fibrosis to a similar degree as in human SSc, confirming their suitability to study STAT3 signaling in SSc (Fig. 3e, f).

**Fig. 4:**
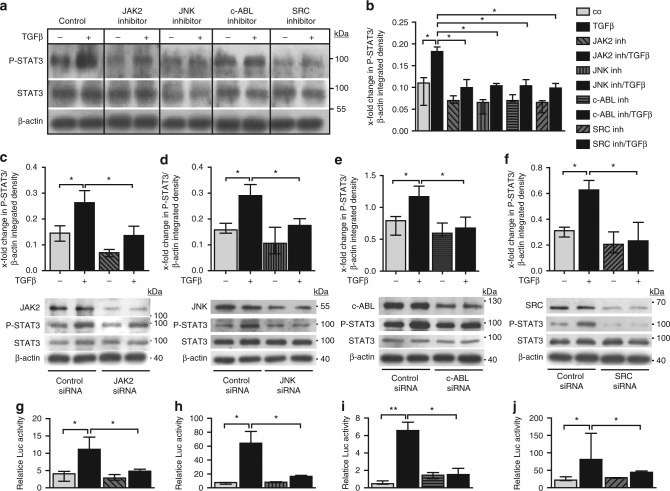
Profibrotic kinases pathways converge on P-STAT3 in vitro. **a**, **b** Effects of pharmacologic inhibition of JAK2, JNK, c-ABL, and SRC kinases on the levels of P-STAT3 in TGFβ-stimulated fibroblasts in vitro as analyzed by **a** western blot and **b** its quantification (*n* ≥ 4 with 2 technical replicates per condition for all experiments). Representative images and quantification of four independent experiments are shown. **c**–**f** Effects of siRNA-mediated knockdown of **c** JAK2, **d** JNK, **e** c-ABL, and **f** SRC, respectively, on levels of P-STAT3 in healthy human dermal fibroblasts stimulated with TGFβ, as shown by representative western blots and quantifications (*n* ≥ 4 with 2 technical replicates per group for all experiments). JAK2 (expected molecular size, 132 kDa), JNK (expected molecular size, 49 and 55 kDa), c-ABL (expected molecular size, 125–135 kDa), and SRC (expected molecular size, 60 kDa) are represented by ladders showing 100, 55, 130, and 70 kDa, respectively. Beta-actin expected molecular weight/size is 42 kDa. **g**–**j** Relative luciferase activity in fibroblasts transfected with STAT3 luciferase reporter plasmid and treated with **g** the JAK2 inhibitor TG101209, **h** the JNK inhibitor SP600125, **i** the c-ABL inhibitor imatinib mesylate and **j** the SRC inhibitor SU6656, independently with or without TGFβ stimulation (*n* = 3 independent experiments with 2 technical replicates per group for all experiments). Luciferase activity was normalized against a non-inducible luciferase construct. Results are shown as median ± interquartile range (IQR). Significance was determined by Mann–Whitney test, as compared to untreated unstimulated fibroblasts or untreated TGFβ-stimulated fibroblasts, respectively. *P < 0.05; **P < 0.01, ***P < 0.001

In vitro studies demonstrate that not only inhibition of JAK2, but also of JNK, SRC, and c-ABL by both siRNA-mediated knockdown and by respective small molecule inhibitors of JAK2, JNK, SRC, and c-ABL all significantly inhibit the TGFβ-induced phosphorylation of STAT3 (Fig. 4a–f). These results are further substantiated by demonstrating that individual inactivation of JAK2, JNK, SRC, and c-ABL all reduced STAT3-dependent reporter activity in TGFβ-stimulated fibroblasts (Fig. 4g–j). Taken together, these findings suggest that JAK2, JNK, SRC, and c-ABL all contribute to the TGFβ-induced activation of STAT3 in fibroblasts.

**Fig. 5:**
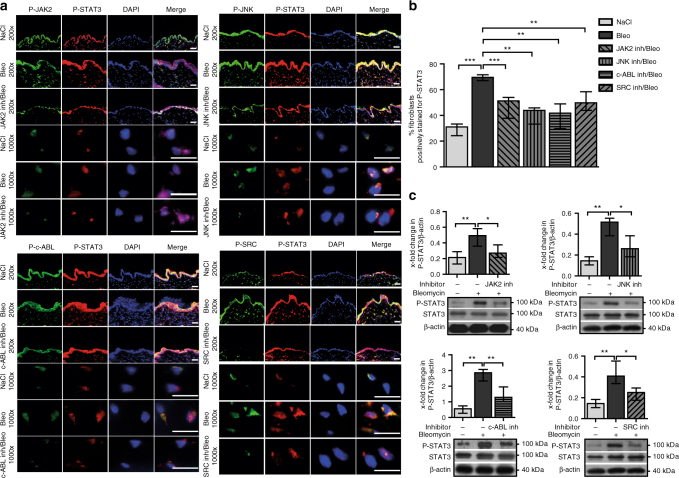
Pharmacological inhibition of JAK2, JNK, c-ABL, and SRC reduces levels of P-STAT3 in bleomycin-challenged mice. **a**–**c a** Representative images with **b** quantification of immunofluorescence stainings and **c** western blot analyses with quantification of P-STAT3 in the skin of bleomycin-challenged mice treated with the JAK2 inhibitor TG101209, the JNK inhibitor CC-930, c-ABL inhibitor imatinib mesylate, or the SRC inhibitor SU6656. Control mice injected with NaCl and bleomycin-challenged receiving vehicle treatment served as controls. *N* ≥ 4 mice and 3 technical replicates per group for all experiments. Expected band size for P-STAT3 and STAT3 are 79 kDa (lower faint band) and 86 kDa (higher intense band) and the ladder represents 100 kDa. Beta-actin (expected molecular weight/size 42 kDa) is shown by ladder at 40 kDa. Magnifications of 200-fold and 1000-fold are shown for all immunofluorescence stainings. Horizontal scale bar, 100 μm. Results are shown as median ± interquartile range (IQR). Significance was determined by Mann–Whitney test, as compared to sham-treated or bleomycin-challenged mice, respectively. *P < 0.05; **P < 0.01, ***P < 0.001

To confirm those findings in vivo, we selectively inhibited JAK2, JNK, SRC, and c-ABL by selective small molecule inhibitors in the experimental murine models of bleomycin-induced skin fibrosis and TBRact-induced skin fibrosis. Individual inhibition of JAK2, JNK, SRC, or c-ABL signaling all significantly ameliorates the bleomycin-induced accumulation of P-STAT3 (Fig. 5a–c). Consistently, treatment with small molecule inhibitors of either JAK2, JNK, SRC, or c-ABL all reduces the accumulation of phosphorylated STAT3 in mice overexpressing TBRact (Supplementary Fig. 2). However, despite relatively high doses of each inhibitor, none of the treatments completely abrogates the activation of STAT3 signaling by bleomycin. These data demonstrate that the activation of STAT3 in activated fibroblasts results from the combined activation of JAK2, JNK, SRC, and c-ABL kinases.

As recent findings suggest that JAK1 might also be capable of activating STAT3, either directly^32,36^ or indirectly by transphosphorylation of JAK2^37^, we decide to further investigate the effects of JAK1 inhibition on TGFβ-induced activation of STAT3. Inhibition of JAK1 by siRNA-mediated knockdown or small molecule inhibitors targeting JAK1 and JAK2 partially reduces STAT3 activation in human dermal fibroblasts (Supplementary Fig. 1).

We are further interested to study potential crosstalk between STAT3 and SMAD signaling in TGFβ-stimulated fibroblasts. We first analyze the kinetics of the activation of both pathways by analyzing the accumulation of P-STAT3 and P-SMAD3 upon stimulation with TGFβ. The accumulation of P-SMAD3 precedes the accumulation of P-STAT3 with initial increases after 15 min for P-SMAD3 and 2–3 h for P-STAT3 (Supplementary Fig. 3a). Co-immunoprecipitation (CoIP) studies with SMAD3 antibodies demonstrate that SMAD3 binds to non-phosphorylated STAT3 in dermal fibroblasts (Supplementary Fig. 3b). The interaction between SMAD3 and non-phosphorylated STAT3 is further enhanced by coincubation of fibroblasts with the STAT3 dimerization inhibitor S3I-201, a small molecule inhibitor that interferes with the dimerization of STAT3^31^, likely by increasing the availability of monomeric STAT3 in the cytoplasm as a binding partner for SMAD3. Incubation with S3I-201, however, does not promote binding of SMAD3 to P-STAT3. Consistent with a recently proposed model that the binding of non-phosphorylated SMAD3 to STAT3 may inhibit STAT3 signaling^36^, siRNA-mediated knockdown of SMAD3 promotes STAT3 signaling with a trend toward increased levels of P-STAT3 and significantly increases STAT3-dependent transcription in reporter assays (Supplementary Fig. 3c–f). However, inhibition of STAT3 dimerization using S3I-201 has no significant impact on TGFβ-induced SMAD3 phosphorylation in cultured fibroblasts and on SMAD3-dependent transcription in reporter studies, suggesting a predominantly unidirectional regulation.

### Inactivation of STAT3 inhibits TGFβ-induced myofibroblast differentiation and collagen release

**Fig. 6:**
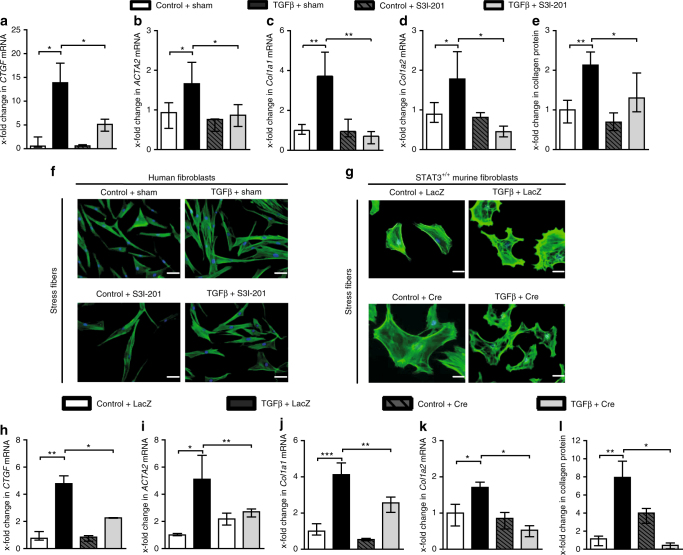
Inactivation of STAT3 inhibits TGFβ-induced myofibroblast differentiation and collagen release. **a**–**f** Effect of the STAT3 inhibitor S3I-201 on the mRNA levels of **a** *CTGF*, **b** *ACTA2*, **c** *COL1A1*, **d** *COL1A2*, and **e** collagen protein (*n* = 4–7 and 2 technical replicates for all experiments) in human dermal fibroblasts. **f** Representative images of immunofluorescence staining for stress fibers at 200-fold magnification. Horizontal scale bar, 100 μm. **g**–**l** Responsiveness of AAV-Cre virus-infected mouse fibroblasts from *STAT3* ^fl/fl^ mice to TGFβ stimulation, as compared to AAV-LacZ virus-infected fibroblasts, which served as control: **g** Representative images of immunofluorescence staining for stress fibers at 200-fold magnification. Horizontal scale bar, 100 μm. mRNA levels of **h** *Ctgf*, **i** *Acta2*, **j** *Col1a1*, **k** *Col1a2*, and **l** collagen protein (*n* = 6 with 2 technical replicates for all experiments). Results are shown as median ± interquartile range (IQR). Significance was determined by Mann–Whitney test, as compared to the unstimulated control fibroblasts or TGFβ-stimulated control fibroblasts, respectively. *P < 0.05; **P < 0.01, ***P < 0.001. AAV adeno-associated virus

We wonder whether the TGFβ-induced activation of STAT3 may contribute to the stimulatory effects of TGFβ on fibroblasts. To target STAT3 signaling, we first use S3I-201, a small molecule inhibitor that binds to the STAT3–SH2 domain to block STAT3 phosphorylation and STAT3 DNA binding^24,38^. Treatment with S3I-201 inhibits the transcription of the TGFβ target gene *Ctgf* and reduces the differentiation of resting fibroblasts into myofibroblasts with impaired upregulation of αSMA mRNA and protein and decreased formation of stress fibers upon stimulation with TGFβ (Fig. 6a–f). Moreover, inhibition of STAT3 also reduces the stimulatory effects of TGFβ on collagen synthesis with decreased mRNA levels of *col1a1* and *col1a2* and reduced release of collagen protein (Fig. 6c–e). To exclude that the anti-fibrotic effects of S3I-201 are due to off-target effects, we confirm these results by a genetic approach using fibroblasts depleted of STAT3. Consistent with the findings obtained with S3I-201, fibroblasts deficient in STAT3 are less sensitive to the stimulatory effects of TGFβ with decreased expression of *Ctgf*, impaired myofibroblast differentiation and reduced collagen synthesis (Fig. 6g–l).

### Fibroblast-specific knockout of STAT3 ameliorates experimental fibrosis

**Fig. 7:**
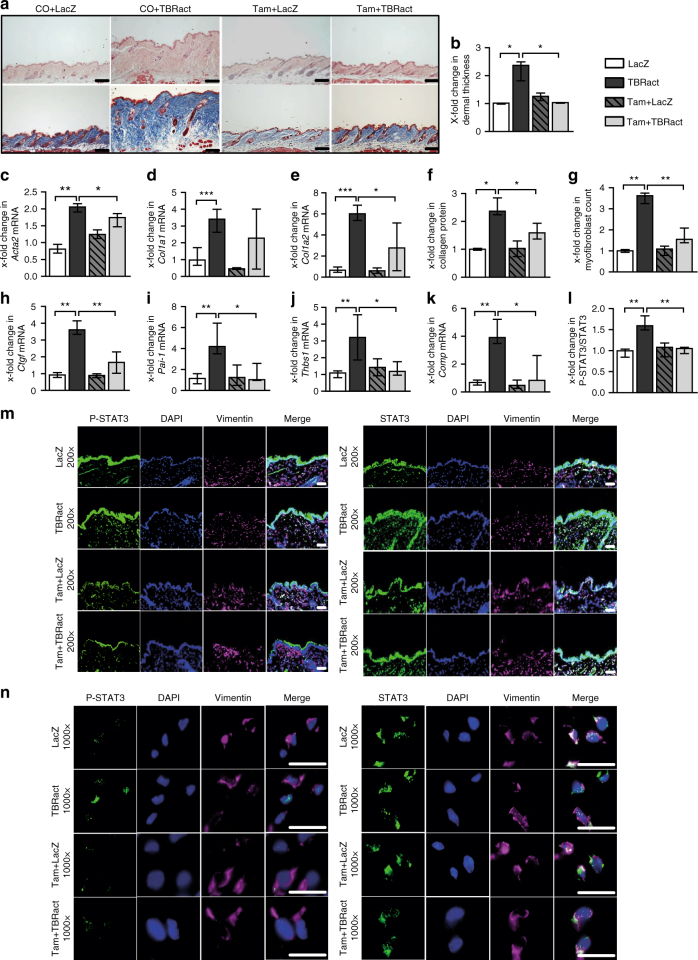
Fibroblast-specific knockout of STAT3 reduced the effects of TBRact-induced skin fibrosis. **a** Representative histological sections stained with hematoxylin and eosin (top) and trichrome (bottom). **b** Dermal thickness, **c**–**e** mRNA levels of *Acta2*, *Col1a1*, and *Col1a2*, respectively, **f** hydroxyproline content, **g** myofibroblast count, and **h**–**k** levels of **h** *Ctgf* mRNA, **i** *Pai-1* mRNA and of the proposed biomarkers, **j** *Thbs1* mRNA and **k** *Comp* mRNA. **m**, **n** Representative immmunofluorescence stainings of P-STAT3 (left) and total STAT3 (right) at 200-fold and 1000-fold magnification, respectively, along with their **l** quantitative analyses. All experiments were performed in dermal tissue sections from the experimental mouse model of TBRact-induced skin fibrosis in mice with fibroblast-specific, tamoxifen-inducible, Cre-loxP-based (*Col1a2-Cre-ER*) knockout of STAT3 in *STAT3* ^fl/fl^ mice and control littermates (C57Bl/6background, 12 weeks of age). *N* ≥ 6 with 2 technical replicates per group for all experiments. Results are shown as median ± interquartile range (IQR). Horizontal scale bar, 100 μm. Significance was determined by Mann–Whitney test, as compared to vehicle-treated, fibrotic mice, respectively. *P < 0.05; **P < 0.01***; *P* < 0.001. Tam tamoxifen; CO, Corn-oil

**Fig. 8:**
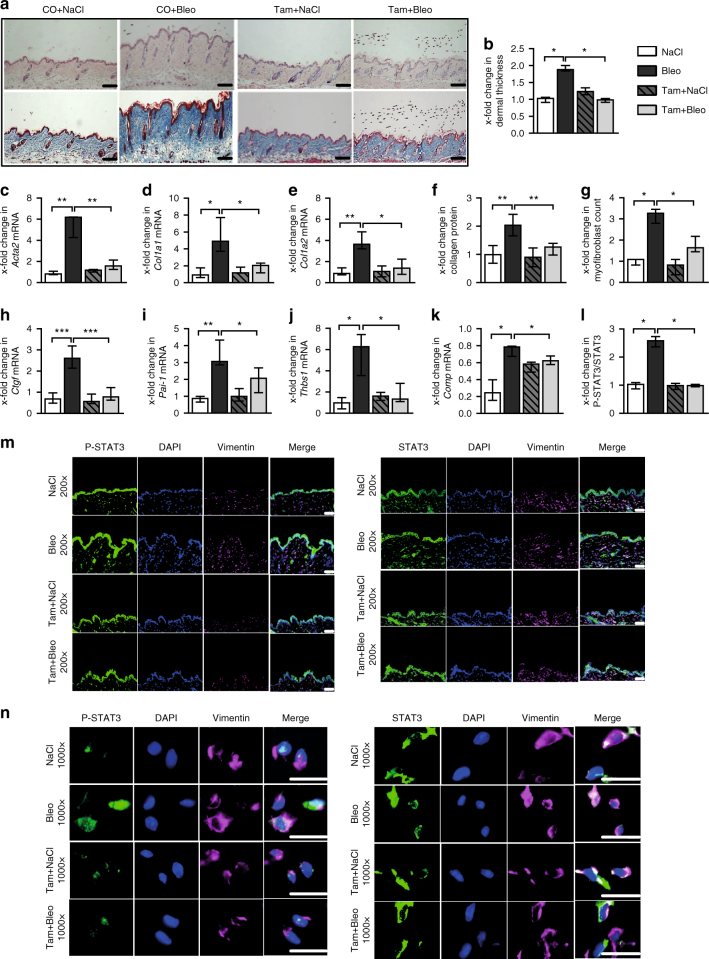
Fibroblast-specific knockout of STAT3 ameliorates bleomycin-induced skin fibrosis. **a** Representative histological sections stained with hematoxylin and eosin (top) and trichrome (bottom). **b** Dermal thickness, **c**–**e** mRNA levels of *Acta2*, *Col1a1*, and *Col1a2*, respectively, **f** hydroxyproline content, **g** myofibroblast count, and **h**–**k** levels of **h** *Ctgf* mRNA, **i** *Pai-1* mRNA and of the proposed biomarkers, **j** *Thbs1* mRNA, and **k** *Comp* mRNA. **m**, **n** Representative immmunofluorescence stainings of P-STAT3 (left) and total STAT3 (right) at 200-fold and 1000-fold magnification, respectively, along with their **l** quantitative analyses. All experiments were performed in the skin tissue sections from the experimental mouse model of bleomycin-induced skin fibrosis in mice with fibroblast-specific, tamoxifen-inducible, Cre-loxP-based (*Col1a2-Cre-ER*) knockout of STAT3 in *STAT3* ^fl/fl^ mice and control littermates (C57Bl/6background, 12 weeks of age). *N* ≥ 6 mice with 2 technical replicates per group for all experiments. Results are shown as median ± interquartile range (IQR). Horizontal scale bar, 100 μm. Significance was determined by Mann–Whitney test, as compared to vehicle-treated, fibrotic mice, respectively. *P < 0.05; **P < 0.01***; *P* < 0.001. Tam tamoxifen, CO Corn-oil

We next investigate whether inactivation of STAT3 exerts anti-fibrotic effects in murine models of SSc. As STAT3 signaling is particularly active in fibroblasts and as mice with complete, non-conditional knockout of STAT3 are not viable^39,40^, we generate mice with selective and inducible deletion of STAT3 in fibroblasts. Knockdown of STAT3 in fibroblasts does not alter the basal skin histology, leukocyte infiltration, or the collagen content in the absence of pro-fibrotic stimuli (Figs 7 and 8 and Supplementary Fig. 4). Furthermore, the fibroblast-specific knockdown of STAT3 in bleomycin-challenged mice does not have any noticeable or adverse effect on STAT3 activation in other cell populations in the murine skin (Supplementary Figs. 5–9).

However, mice with conditional deletion of STAT3 in fibroblasts are protected from experimental fibrosis. Consistent with the inhibitory effects of STAT3 inactivation on TGFβ-induced fibroblast activation in vitro, mice lacking STAT3 selectively in fibroblasts are less sensitive to TBRIact-induced fibrosis and demonstrate reduced dermal thickening, decreased myofibroblast counts, and lower hydroxyproline levels compared to TBRIact mice with normal STAT3 expression (Fig. 7a–g). Consistent with decreased myofibroblast counts and the reduced collagen deposition, the mRNA levels of *Acta2*, *Col1a1*, and *Col1a2* are also reduced in mice with fibroblast-specific deletion of STAT3-overexpressing TBRIact as compared to control littermates (Fig. 7c–e).

The mRNA levels of the TGFβ-regulated genes *Thbs1* and *Comp* have recently been shown to correlate with the changes in the modified Rodnan skin score^41–44^ as the common clinical readout of skin fibrosis and changes in the mRNA levels of *Thbs1* and *Comp* in fibrotic skin are thus considered as potential biomarkers for SSc. The mRNA levels of *Thbs1* and *Comp* decrease in mice with fibroblast-specific deletion of STAT3 (Fig. 7j, k). The mRNA levels of other TGFβ-regulated genes, such as *Pai-1* and *Ctgf*, are also found to be reduced (Fig. 7h, i).

In addition to TBRIact-induced fibrosis, mice with fibroblast-specific knockout of STAT3 are also protected from bleomycin-induced skin fibrosis with reduced dermal thickening, myofibroblast differentiation, and hydroxyproline content in lesional skin compared to control mice (Fig. 8a–g). The levels of *Acta2*, *Col1a1*, *Col1a2, Thbs1*, *Comp*, *Pai-1*, and *Ctgf* mRNA lessen in mice with fibroblast-specific deletion of STAT3 upon challenge with bleomycin than in control littermates (Fig. 8h–n).

### Pharmacological inhibition of STAT3 exerts potent anti-fibrotic effects in experimental models of SSc

**Fig. 9:**
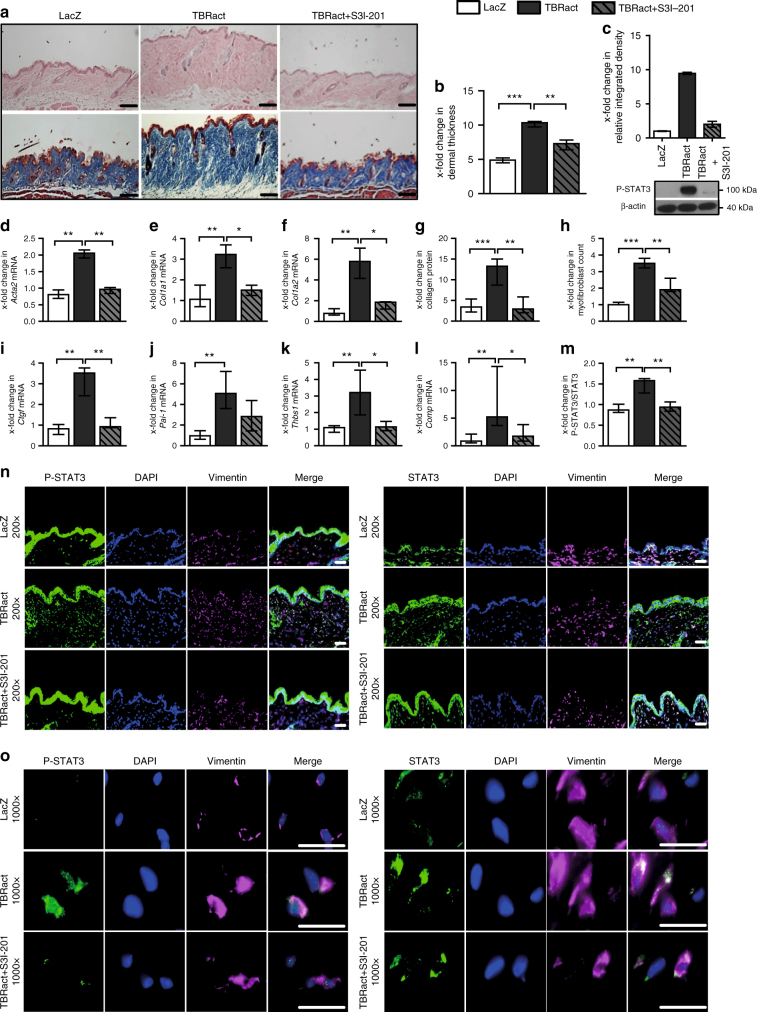
Pharmacological inhibition of STAT3 induces the regression of TBRact-induced experimental skin fibrosis. **a**–**o** Treatment of TBRact-induced skin fibrosis with the STAT3 inhibitor S3I-201 in mice (DBA/2 background, 12 weeks of age). **a** Representative histological sections stained with hematoxylin and eosin (top) and trichrome (bottom). **b** Dermal thickness, **c** western blot analysis of P-STAT3, shown by the ladder representing 100 kDa (expected intense upper band size, 86 kDa and lower faint band size, 79 kDa). Beta-actin (expected molecular weight/size, 42 kDa) is shown by ladder at 40 kDa. **d**–**f** mRNA levels of **d** *Acta2*, **e** *Col1a1*, and **f** *Col1a2*, **g** hydroxyproline content, **h** myofibroblast counts and levels of **i** *Ctgf* mRNA, **j** *Pai-1* mRNA and of the proposed biomarkers, **k** *Thbs1* mRNA, and **l** *Comp* mRNA. **m**–**o** Immunofluorescence analysis including **n**, **o** representative immunofluorescence stainings of P-STAT3 (left) and total STAT3 (right) at 200-fold and 1000-fold magnification, respectively, along with their **m** quantitative analyses. *N* ≥ 6 mice with 2 technical replicates per group for all experiments. Results are shown as median ± interquartile range (IQR). Horizontal scale bar, 100 μm. Significance was determined by Mann–Whitney test, as compared to vehicle-treated, fibrotic mice, respectively. *P < 0.05; **P < 0.01***; *P* < 0.001

**Fig. 10:**
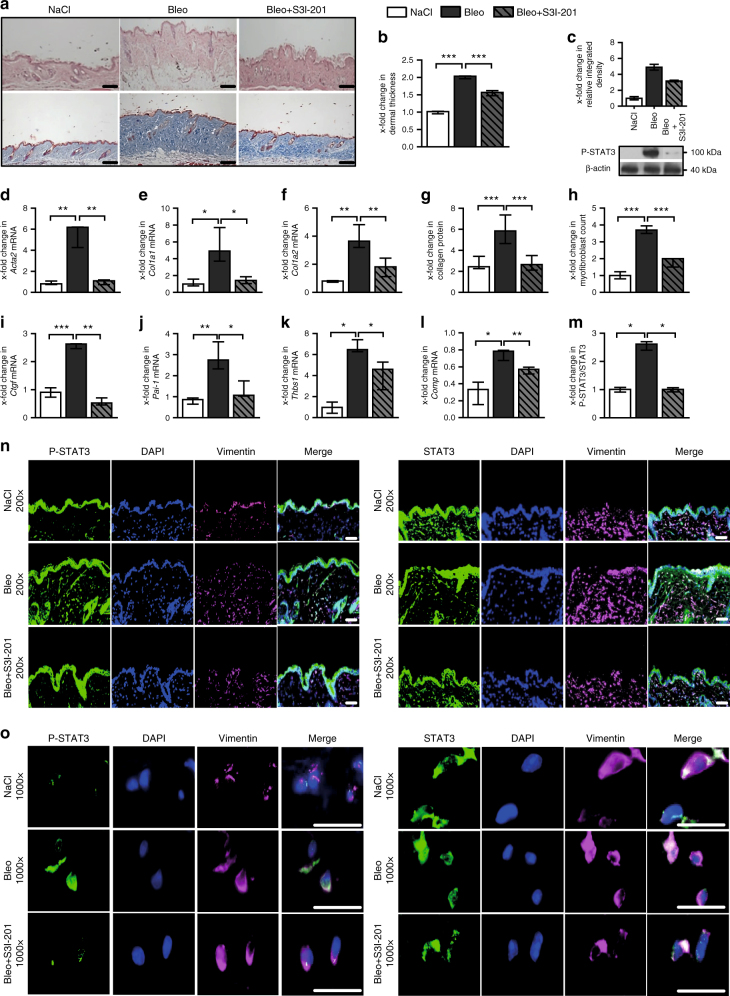
Pharmacological inhibition of STAT3 exerts potent anti-fibrotic effects in bleomycin-induced experimental skin fibrosis model. **a**–**o** Treatment of bleomycin-induced skin fibrosis with the STAT3 inhibitor S3I-201 in mice (DBA/2 background, 12 weeks of age). **a** Representative histological sections stained with hematoxylin and eosin (top) and trichrome (bottom). **b** Dermal thickness, **c** western blot analysis of P-STAT3, shown by the ladder representing 100 kDa (expected intense upper band size, 86 kDa and lower faint band size, 79 kDa). Beta-actin (expected molecular weight/size, 42 kDa) is shown by ladder at 40 kDa. **d**–**f** mRNA levels of **d** *Acta2*, **e** *Col1a1*, and **f** *Col1a2*, **g** hydroxyproline content, **h** myofibroblast counts and levels of **i** *Ctgf* mRNA, **j** *Pai-1* mRNA and of the proposed biomarkers, **k** *Thbs1* mRNA, and **l** *Comp* mRNA. **m**–**o** Immunofluorescence analysis including **n**, **o** representative immmunofluorescence stainings of P-STAT3 (left) and total STAT3 (right) at 200-fold and 1000-fold magnification, respectively, along with their **m** quantitative analyses. *N* ≥ 6 mice with 2 technical replicates per group for all experiments. Results are shown as median ± interquartile range (IQR). Horizontal scale bar, 100 μm. Significance was determined by Mann–Whitney test, as compared to vehicle-treated, fibrotic mice, respectively. *P < 0.05; **P < 0.01***; *P* < 0.001

To test the translational potential of those findings, we next use S3I-201 to inactive STAT3 in experimental fibrosis. Treatment with S3I-201 exerts potent anti-fibrotic effects in TBRIact- and bleomycin-induced skin fibrosis at well-tolerated doses (Figs. 9 and 10). S3I-201 effectively reduces TBRIact-induced dermal thickening, myofibroblast differentiation, and collagen accumulation (Fig. 9a–h).

Treatment with S3I-201 also strongly ameliorates bleomycin-induced skin fibrosis (Fig. 10a–h). In both models, treatment with S3I-201 reduces the mRNA levels of *Acta2*, *Col1a1*, *Col1a2, Thbs1*, *Comp*, *Pai-1*, and *Ctgf* (Figs. 9 and 10i–l).

Of note, no evidence of toxicity of anti-fibrotic doses of S3I-201 has been observed, either clinically or on necroscopy.

## Discussion

In this study, we characterize STAT3 as an intracellular mediator of the pro-fibrotic effects of TGFβ. We show that knockdown of STAT3 in cultured fibroblasts by siRNA or by pharmacological inactivation prevents TGFβ-induced differentiation of resting fibroblast into myofibroblasts and significantly reduces the stimulatory effect of TGFβ on collagen release. Moreover, fibroblast-specific knockout of STAT3 strongly ameliorates TBRact-induced fibrosis, further highlighting the crucial role of STAT3 in TGFβ-induced fibroblast activation. We establish that pharmacological or genetic inhibition of STAT3 also reduces the levels of TGFβ-target genes in skin experimental fibrosis, including those of potential biomarkers such as *Thbs1* and *Comp*. TGFβ also activates STAT3 signaling and promotes accumulation of P-STAT3. The stimulatory effects of TGFβ on STAT3 are not restricted to fibroblasts and the skin, but have also been observed in other cell types and tissues such as follicular T-helper cells and hepatic stellate cells^32,45–47^. Together, these data demonstrate that STAT3 acts as a non-canonical downstream mediator to transmit the profibrotic effects of TGFβ.

Although JAK2 is a key regulator of STAT3 phosphorylation, several other kinases have also been shown to phosphorylate and activate STAT3 in a JAK2-independent manner^16,19,48,49^. As inhibition of JAKs would not block those alternative pathways of STAT3 phosphorylation, targeting of JAK2 may thus not completely abrogate the pathological activation of STAT3 in fibrotic diseases. Indeed, we elucidate that STAT3 is phosphorylated not only by JAK2, but that SRC, c-ABL, and JNK kinases also contribute significantly to the phosphorylation of STAT3 in cultured fibroblasts and in fibrotic tissues. In addition, JAK1 also seems to contribute to activation of STAT3 in fibroblasts in response to TGFβ, but further studies are required to determine whether JAK1 directly phosphorylates STAT3 in this context or whether the effects or mediated indirectly via JAK1-dependent activation of JAK2^32,36,37^. These findings demonstrate that the signals transmitted through JAK2, SRC, c-ABL, and JNK all converge on STAT3. This is particularly intriguing as all these kinases are hyper-activated in SSc and have been shown to contribute to the aberrant activation of fibroblasts^13,34,35,50^. In addition to these kinases, serine/threonine kinases, such as CK2, have also been implicated in the pathogenesis of SSc^36^. Whether CK2 and other serine/threonine kinases are also capable of activating STAT3 signaling remains to be determined. These data provide evidence that STAT3 serves as a key molecular checkpoint for fibroblast activation by integrating and converting activation of JAK2, SRC, c-ABL, and JNK kinases into pro-fibrotic responses with induction of myofibroblast differentiation and upregulation of collagen release.

We also investigate the crosstalk of STAT3 signaling with canonical TGFβ/SMAD signaling. In some contexts, STAT3 and SMAD3 signaling pathways have been reported complementary to each other^32,36^, whereas recent data generated in HaCaT cells suggest that STAT3 signaling may also attenuate TGFβ-induced SMAD signaling^51^. We demonstrate that the activation of SMAD3 signaling precedes the activation STAT3 signaling in primary human dermal fibroblasts. Moreover, in this setting, SMAD3 binds to non-phosphorylated STAT3, but not to P-STAT3. Knockdown of SMAD3 enhances TGFβ-induced STAT3 signaling, whereas inhibition of STAT3 does not promote SMAD signaling. These findings provide evidence that the binding of non-phosphorylated SMAD3 to STAT3 may inhibit STAT3 signaling in fibroblasts in the context of fibrosis, a model that is consistent with recent findings in hepatic stellate cells^32,36^. Activation of SMAD signaling in fibrotic diseases may thus further boost TGFβ-dependent activation of STAT3 in addition to direct phosphorylation by the upstream kinases SRC, JAK2, c-ABL, and JNK.

The central role of STAT3 as an integrator of pro-fibrotic signals from several upstream kinases suggests STAT3 as a potential target for anti-fibrotic therapies. Indeed, we demonstrate using genetic approaches as well as a selective pharmacological inhibition of STAT3 that inactivation of STAT3 signaling exerts potent anti-fibrotic effects. Inactivation of STAT3 inhibits bleomycin-induced fibrosis as a model of early, inflammation-driven fibrosis, but also demonstrate potent anti-fibrotic effects in TBRact-induced fibrosis as a model of late, non-inflammatory stages of SSc with endogenous activation of fibroblasts^52^. Targeting STAT3 may thus be effective in different stages and different subpopulation of SSc patients. Moreover, treatment with S3I-201 is not only effective in preventive settings, but also in therapeutic regimens. When treatment with S3I-201 is initiated after fibrosis has already been established, inhibition of STAT3 does not only prevent further progression of fibrosis despite ongoing challenge with bleomycin, but induces regression of fibrosis to below-pre-treatment levels.

Although our in vitro studies on cultured fibroblasts and our in vivo data on mice with ablation of STAT3 specifically in fibroblasts demonstrate that fibroblasts are key-target cells, the anti-inflammatory effects of STAT3 inhibitors likely contribute to the anti-fibrotic effects of pharmaceutical STAT3 inhibition. STAT3 transmits the intracellular signals of multiple cytokines, such as IL-1β, IL-6, or TNFα, that are known to promote inflammation in fibrotic diseases including SSc^26,27,53–55^. Inhibition of IL-6 signaling may be of particular interest for the pathogenesis of SSc, as treatment with anti-IL6-receptor antibodies may improve the clinical outcome in an inflammatory subgroup of SSc patients^27,56^. Although approximately only one-third of SSc patients shows an inflammatory gene expression profile in fibrotic skin or presents with clinical features of inflammation^57–60^, inhibition of inflammation may contribute to the efficacy of STAT3 inhibitors in particular in the inflammatory subset of patients. For the same reasons, inhibition of STAT3 may not only be effective in classical fibrotic diseases, but may also ameliorate pathologic repair responses in inflammatory diseases. For example, treatment with Stattic, a first-generation inhibitor of STAT3, also improves strictures in Crohn’s diseases^61^; treatment with a small STAT3 inhibitor STA-21, ameliorates psoriasis-like skin lesions not only in experimental mouse models, but may also improve human psoriasis^21,53,62^. The prominent role of STAT3 in the pathogenesis of psoriasis is intriguing, i.p. as we also observe staining for P-STAT3 in keratinocytes in our mouse models. However, the levels of nuclear P-STAT3 in epidermal keratinocytes are not altered by induction of fibrosis with bleomycin (Supplementary Fig. 10 and Supplementary Movies 1 and 2).

These findings may have direct translational implications. Several approaches to target STAT3 are currently in clinical development with small molecular inhibitors of STAT3 dimerization and oligodeoxynucleotides being most common. Indeed, more than 10 different clinical trials investigating STAT3 inhibitors in various solid tumors, hematologic neoplasms, or psoriasis are currently ongoing or have already been completed (www.clinicaltrials.gov), highlighting that STAT3 is considered as a prime target for pharmaceutical intervention.

## Methods

### Patients

Dermal fibroblasts were isolated from skin biopsies of 27 SSc patients fulfilling the criteria for SSc^63^ (Supplementary Table 1) and from skin biopsy samples of 31 healthy individuals matched for age and sex as described. Clinical characteristics are provided in Supplementary Table 1. The human studies were approved by the Ethical Committee of the Medical Faculty of the University of Erlangen-Nuremberg. All patients and controls signed a consent form approved by the local institutional review board.

All patients had SSc according to the 2013 ACR/EULAR^64^. The disease subset was determined according to the criteria proposed by LeRoy et al. Disease duration was measured from the onset of the first non-Raynaud symptoms attributable to SSc. Pulmonary arterial hypertension was diagnosed by right heart catheterization. Disease activity was determined using the EULAR Systemic Sclerosis Activity Score. Patients with scores of ≥3 were classified as having active disease. DMARD, disease-modifying antirheumatic drug; F, female; M, male; NSAID, non-steroidal anti-inflammatory drug.

### Cell culture

Murine and human dermal fibroblasts were obtained from sterile 3 mm × 3 mm skin punches from murine or patients. The skin fragments were further fragmented using sterile scalpel and digested with Dispase II solution (from Bacillus polymyxa, Gibco BRL, Darmstadt, Germany) (0.8 mg/ml in PBS) for 3 h at 37 °C and 800 rpm. The digested sample was filtered using 100 µm nylon filter and centrifuged at 1400 rpm for 5 min. The pellet was resuspended in Dulbecco’s modified Eagle’s medium-Ham’s F-12 (DMEM) containing 10% fetal bovine serum and put in culture flasks. Before an experiment, dermal fibroblasts were serum-starved in DMEM containing 0.1% fetal bovine serum for 24–48 h. In selective experiments, serum-starved cells were incubated with recombinant human TGFβ-1 (10 ng/ml) (R&D Systems, Abingdon, UK), the STAT3 inhibitor S3I-201 (15 µM) (Selleckchem, Houston, USA), the JAK2 inhibitor TG101209 (500 nM) (Selleckchem), the JAK1/2 inhibitor Ruxolitinib (5 µM) (LC Laboratories, Massachusetts, USA), the SRC inhibitor SU6656 (500 nM) (Calbiochem, Seattle, USA), the JNK inhibitor SP600125 (500 nM) (Tocris Bioscience, Bristol, UK), and the c-ABL inhibitor imatinib mesylate (500 nM) (Novartis, Basel, Switzerland). To delete STAT3 from cultured murine fibroblasts isolated from STAT3^fl/fl^ Cre^−/−^ mice, fibroblasts were infected with type 5 adeno-associated viruses (AAV) encoding for Cre recombinase at an IFU of 80/cells. AAV encoding for LacZ served as controls.

### Transfections and luciferase reporter assays

Human dermal fibroblasts were transfected with reporter plasmid constructs or siRNAs using using the Nucleofection technique and Nucleofector Solution V (Lonza, Cologne, Germany) using the manufacturer’s recommended protocol. Experiments were conducted 24–48 h after transfection and thereafter cells were harvested. Gene silencing was achieved by nucleofecting 3 μg pre-designed siRNA duplexes against *JAK2*, *JAK1*, *SRC*, *c-ABL*, and *SMAD3* (all Eurogentec, Seraing, Belgium) and 100 nM SignalSilence siRNA I for SAPK/JNK (Cell Signaling Technology, #6232). The transfection efficiency was determined by western blot analysis. Non-targeting siRNAs (nt siRNA) (Life Technologies, Darmstadt, Germany) served as controls. The sequences of the pre-designed sense siRNAs are described in Supplementary Table 2.

Cignal Lenti STAT3 Reporter (luc) Kit and Cignal SMAD Reporter (luc) Kit from Qiagen (Hilden, Germany) were used to determine STAT3- and SMAD3-dependent reporter activities. Dual-luciferase activities were determined by using Luminoskan™ Ascent Microplate-Luminometer (ThermoFisher Scientific, Madrid, Spain). The constitutively expressed non-inducible Renilla luciferase activity served as internal control for normalizing transfection efficiencies.

### Quantitative real-time PCR

Gene expression was quantified by SYBR Green real-time PCR using the ABI Prism 7300 Sequence Detection System (Life Technologies). Samples without enzyme in the reverse transcription reaction (non-RT controls) were used as negative controls. Unspecific signals caused by primer dimers were excluded by non-template controls and by dissociation curve analysis. Beta-actin (*ACTB*) was used to normalize for the amounts of cDNA within each sample. All primer sequences are presented in Suppplementary Table 3.

### Western blot analysis

Proteins were separated by SDS-PAGE and transferred to polyvinylidene difluoride membrane, which was incubated overnight with the appropriate primary antibody. The primary antibodies included mouse anti-STAT3 (1:1000), rabbit anti-P-STAT3 (Tyr705, dilution 1:500), rabbit anti-JAK2 (dilution 1:1000), rabbit anti-P-SRC (Tyr 416, dilution 1:500), rabbit anti-SRC (dilution 1:800), rabbit anti-c-ABL (dilution 1:800), rabbit anti P-c-ABL (Tyr 245/412, dilution 1:500), rabbit and anti-JNK (SAPK/JNK, dilution 1:500) from Cell Signaling Technology (Frankfurt, Germany); rabbit anti-JAK1 (1:200), goat anti-P-JAK1 (Tyr 1022, dilution 1:200), and rabbit anti-P-JAK2 (Tyr 1007/1008, dilution 1:200) from Santa Cruz Biotechnology (Heidelberg, Germany); rabbit anti-SMAD3 (dilution 1:500), rabbit anti-P-SMAD3 (dilution 1:250), and rabbit anti-P-JNK (Thr183/Tyr185, dilution 1:500) from Abcam (Cambridge, UK) and rabbit anti-P-SRC (dilution 1:500) from MyBioSource (San Diego, USA). Horseradish peroxidase-conjugated antibodies (Dako, Hamburg, Germany) were used as secondary antibodies. Blots were visualized using enhanced chemiluminescence (ECL from GE Healthcare, Braunschweig, Germany). Beta-actin (Sigma-Aldrich, Deisenhofen, Germany) or Lamin A/C (Cell Signaling Technology) antibodies served as controls for equal loading. PageRuler™ Prestained Protein Ladders #26616 and #26619 from ThermoFisher Scientific (Darmstadt, Germany) were used. Bands were quantified using the ImageJ Software (NIH, version 1.49). The original uncropped scans of western blots presented in the main figures are shown in Supplementary Fig. 11.

### CoIP

Fibroblasts were collected in lysis buffer composed of 400 mM NaCl, 20 mM HEPES (pH 7.9), and 1 mM EDTA. An aliquot of 20 μg from the lysates was used as input. Cell extracts were incubated with 20 μl Protein A/G Sepharose and 3 μg of SMAD3 or normal IgG antibodies (no. sc-5569, no. sc-101154, and no. sc-2027, all Santa Cruz Biotechnology, Heidelberg, Germany). Unbound proteins were removed by washing with 0.05% NP-40. Sepharose-bound protein complexes were analysed by western blotting.

### Quantification of collagen protein

Total soluble collagen in cell culture supernatants was quantified using the SirCol collagen assay (Biocolor, Belfast, Northern Ireland) as described previously^50,65^. Briefly, cell culture supernatant was mixed with sirius red dye for 30 min at room temperature. After centrifugation, the pellet was dissolved in alkali reagent. Measurement was performed using a SpectraMax 190 microplate spectrophotometer (Molecular Devices, Biberach an der Riß, Germany) at a wavelength of 540 nm.

### Immunohistochemistry and immunofluorescence staining

Formalin-fixed, paraffin-embedded skin sections or 4% PFA-fixed, 0.25% Triton X-100-permeabilized cells were stained with appropriate primary antibodies, including mouse anti-STAT3 (dilution 1:200) (Cell Signaling Technology) and rabbit anti-P-STAT3 (dilution 1:500) (Abcam). For double-staining experiments, the samples were incubated again with primary antibodies against P-SRC (dilution 1:500), P-JAK2 (dilution 1:500), P-c-ABL (dilution 1:300), and P-JNK (dilution 1:500). Fibroblasts were stained specifically with vimentin (Sigma-Aldrich) overnight at 4 °C. HRP-conjugated or Alexa Fluor antibodies (Life Technologies, Darmstadt, Germany) were used as secondary antibodies. Fibroblasts incubated with isotype control antibodies (Santa Cruz Biotechnology, Heidelberg, Germany) were used as controls. Counter staining of cell nuclei was performed using DAPI (Santa Cruz Biotechnology). Stained cells were visualized using a Nikon Eclipse 80i microscope (Nikon).

For quantification, single, spindle-shaped cells in the dermis positive for the required positive antibody were counted in six randomly chosen high-power fields at 200-fold magnification by two experienced researchers in a blinded manner as described^13^.

Integrated density was analyzed using ImageJ Software (NIH, version 1.49).

### Confocal microscopy and analysis

Confocal images of tissue sections were acquired using a Leica SP5 II confocal laser scanning microscope (Leica Microsystems, Heidelberg, Germany) with ×63 glycerol-immersion objective and scanning resolution of 512 × 512 pixels, zoom factor 6.4. Image stacks consisting of a series of images at 1 μm intervals throughout the entire cell nucleus were taken at randomly selected tissue areas. The images were deconvoluted with the Huygens deconvolution software (Scientific Volume Imaging B.V.). The DAPI channels were then smoothed by convolving with a Gaussian kernel (sigma = 5 pixels) and nuclei were segmented by automatic global thresholding (using ImageJ’s Otsu method). Likewise, YY in the XX channel were segmented using automatic Otsu global thresholding. Afterwards, the ratios of YY between inside and outside of nuclei were calculated for each image. The entire workflow was performed fully automatically to exclude any bias, by a custom ImageJ macro.

### Animal studies

Two different mouse models were employed: bleomycin-induced skin fibrosis, and fibrosis induced by injections of replication-deficient type 5 adenoviruses encoding for a constitutively active TBRI construct^52,66^. For bleomycin-induced skin fibrosis, 6-week-old mice received repeated subcutaneous injections of bleomycin (100 µl) at a concentration of 0.5 mg/ml in defined areas of 1 cm^2^ at the upper back every other day for 4 weeks. Mice injected with equal volumes of 0.9 % sodium chloride served as controls. In a subset of experiments, mice were treated with intraperitoneal injection of c-ABL inhibitor imatinib mesylate (150 mg/kg/day) or SRC inhibitor SU6656 (12 mg/kg/day dissolved in 20% DMSO/80% NaCl) or an oral gavage of JAK2 inhibitor TG101209 (200 mg/kg/day) or JNK inhibitor CC-930 (300 mg/kg/day, Celgene, New Jersey, USA) for 21 days. Control mice were injected with bleomycin and with vehicle for the respective individual inhibitors. All inhibitors were dissolved in 0.9% NaCl unless otherwise mentioned. For TBRact-induced fibrosis, 4-week-old mice received of 6.67 × 10^7^ pfu/mouse of replication-deficient type 5 adenoviruses encoding for TBRI^act^ into defined areas of 1 cm^2^ at the upper back four times per 2 months. Mice injected with LacZ-expressing viruses served as controls. To selectively inactivate STAT3 in fibroblasts, mice carrying two conditional alleles of STAT3 (*STAT3* ^fl/fl^) were crossbred with *col1a2-Cre-ER* mice to generate *col1a2-Cre-ER STAT3* ^fl/fl^. Cre-mediated recombination was induced by repeated i.p. injections of tamoxifen over 5 days. Control groups were injected with corn oil. For pharmacological inhibition of STAT3, we used selective STAT3 inhibitor S3I-201 (10 mg/kg/day). All mouse experiments were approved by the governments of Mittelfranken and/or Unterfranken.

### Histologic analysis

The injected skin areas of all mice were fixed in 4% formalin and embedded in paraffin. Histologic sections were stained with hematoxylin and eosin for the determination of dermal thickness. The dermal and hypodermal thickness was visualized using a Nikon Eclipse 80i microscope (Nikon) and analyzed at four different sites in each mouse in a blinded manner as described^13,65^. For visualization of collagen content, trichome staining was performed (Sigma-Aldrich). Hydroxyproline content and α-smooth muscle actin positive myofibroblasts were analyzed as described previously^65,67^.

### Statistics

All data are presented as median with interquartile range (IQR). Differences between the groups were tested for their statistical significance by Mann–Whitney *U*-test for non-related samples and by the Wilcoxon signed rank tests for related samples. In a subset of experiments, the mean values of the control groups were set to 1. All other values were expressed as x-fold changes compared with the respective controls used as ‘comparison mean values’. *P* values less than 0.05 were considered significant.

### Data availability

All data generated or analysed during this study are included in this published article (and its Supplementary Information files). Additional supporting informations are available from the corresponding author on reasonable request.

## Electronic supplementary material

Supplementary Information

Description of Additional Supplementary Files

Supplementary Movie 1

Supplementary Movie 2
