# Author Correction: Activation of STAT3 integrates common profibrotic pathways to promote fibroblast activation and tissue fibrosis

**DOI:** 10.1038/s41467-021-27450-x

**Published:** 2021-12-08

**Authors:** Debomita Chakraborty, Barbora Šumová, Tatjana Mallano, Chih-Wei Chen, Alfiya Distler, Christina Bergmann, Ingo Ludolph, Raymund E. Horch, Kolja Gelse, Andreas Ramming, Oliver Distler, Georg Schett, Ladislav Šenolt, Jörg H. W. Distler

**Affiliations:** 1 Department of Internal Medicine 3 –Rheumatology and Immunology, Friedrich-Alexander-University Erlangen-Nürnberg (FAU) and University Hospital Erlangen, Erlangen, 91054 Germany; 2 Institute of Rheumatology and Department of Rheumatology, First Faculty of Medicine, Charles University, Prague, 120 00 Czech Republic; 3 Department of Plastic and Hand Surgery and Laboratory for Tissue Engineering and Regenerative Medicine, University Hospital of Erlangen, Friedrich-Alexander University of Erlangen-Nürnberg (FAU), Erlangen, 91054 Germany; 4 Department of Orthopaedic Trauma Surgery, University Hospital Erlangen, Friedrich-Alexander University of Erlangen-Nürnberg (FAU), Erlangen, 91054 Germany; 5 Center of Experimental Rheumatology and Zurich Center of Integrative Human Physiology, University Hospital Zurich, Zurich, 8091 Switzerland

Correction to: *Nature Communications* 10.1038/s41467-017-01236-6, published online 24 October 2017.

The original version of this article contained errors in Fig. 4a and Fig. 10o, which occurred during image assembly. In Fig. 4a, the representative Western blot images for STAT3 under c-ABL inhibitor were a duplication of those under SRC inhibitor. The correct version of Fig. 4a is:
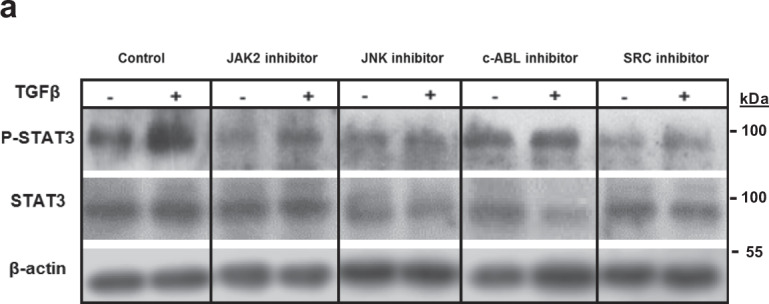


In Fig. 10o, the staining series for NaCl and Bleo in the set of panels to the right was a duplication of the panels for NaCl and Bleo in Fig. 8n. The correct version of Fig. 10o is:
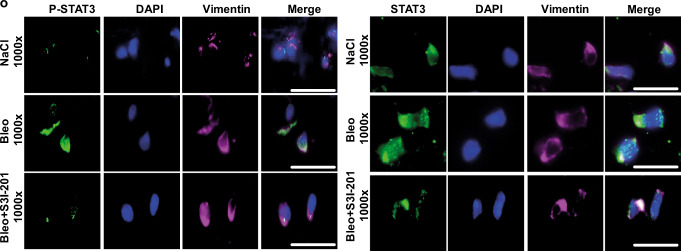


These errors have not been corrected in the PDF and HTML versions of the article.

