# Supplementary material for: Activation of STAT3 integrates common profibrotic pathways to promote fibroblast activation and tissue fibrosis
Source: Nat Commun. 2017 Oct 24;8:1130. doi: 10.1038/s41467-017-01236-6 (PMC5654983; doi:10.1038/s41467-017-01236-6)
Supplement: Supplementary file 1 — Supplementary Information [file 41467_2017_1236_MOESM1_ESM.pdf]

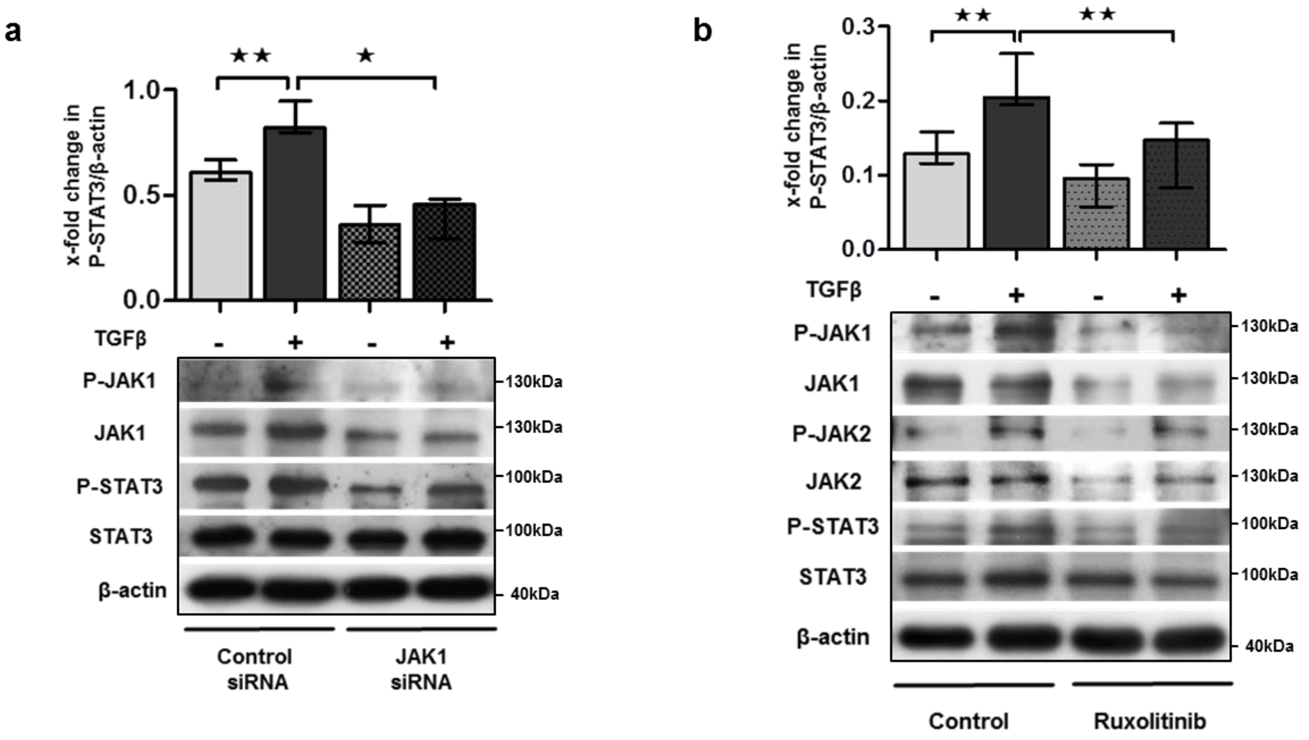

Supplementary Figure 1: JAK1 inhibition reduces STAT3 activation in fibroblasts.

(a;b) Representative Western blot and quantification showing levels of STAT3 phosphorylation in cultured human dermal fibroblasts in response to TGFβ stimulation, upon inhibition of JAK1 mediated by **(a)** siRNA knockdown and **(b)** small molecule repressor Ruxolitinib. Expected band size for P-JAK1 and JAK1 are 133kDa and 130 kDa respectively and the ladder represents 130 kDa. Expected band size for P-STAT3 and STAT3 are 79 kDa (lower faint band) and 86 kDa (higher intense band) and the ladder represents 100kDa. Beta-actin expected molecular weight/size is 42 kDa and the ladder represents 40 kDa. n ≥ 4 independent cell-lines with 2 technical replicates per group for all experiments. Results are shown as median ± interquartile range (IQR). *p<0.05, **p<0.01 versus healthy dermal fibroblasts or TGFβ-stimulated fibroblasts with vehicle, respectively.

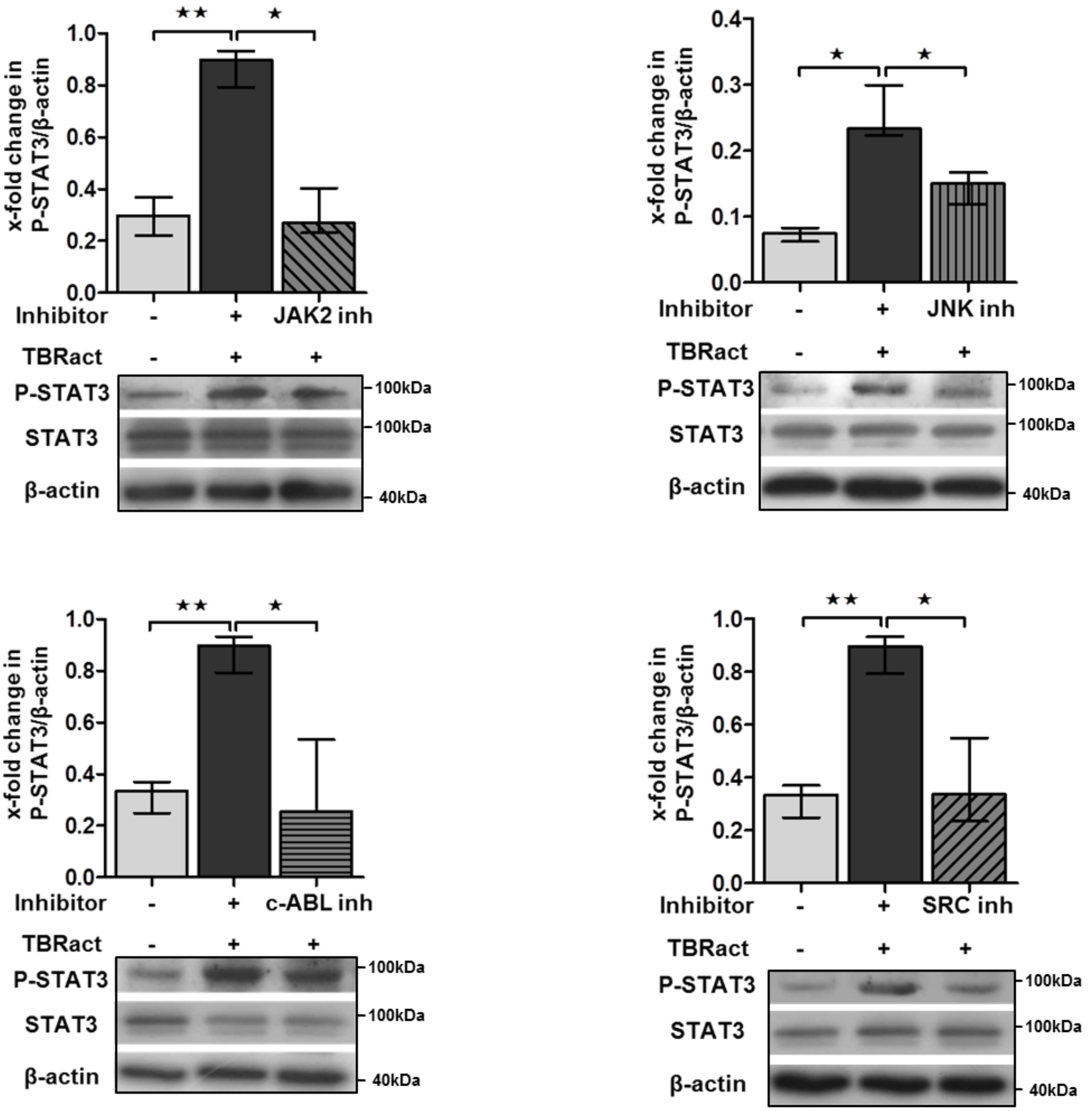

Supplementary Figure 2: Reduction of P-STAT3 upon pharmacological inhibition of JAK2, JNK, c-ABL and SRC in TBRact- induced experimental skin fibrosis.

Western blot analyses with quantification of P-STAT3 in the skin of mice treated with the JAK2 inhibitor TG101209, the JNK inhibitor CC-930, c-ABL inhibitor imatinib mesylate or the SRC inhibitor SU6656, respectively, in the settings of TBRact-induced experimental fibrosis. Expected band size for P-STAT3 and STAT3 are 79 kDa (lower faint band) and 86 kDa (higher intense band) and the ladder represents 100kDa. Beta-actin expected molecular weight/size is 42 kDa. $n \geq 4$ for all mouse groups with 2 technical replicates for all experiments. Results are shown as median ± interquartile range (IQR). Significance was determined by Mann-Whitney test, as compared to vehicle-treated mice overexpressing TBRact or non-fibrotic LacZ mice, respectively. *$p<0.05$; **$p<0.01$, ***$p<0.001$.

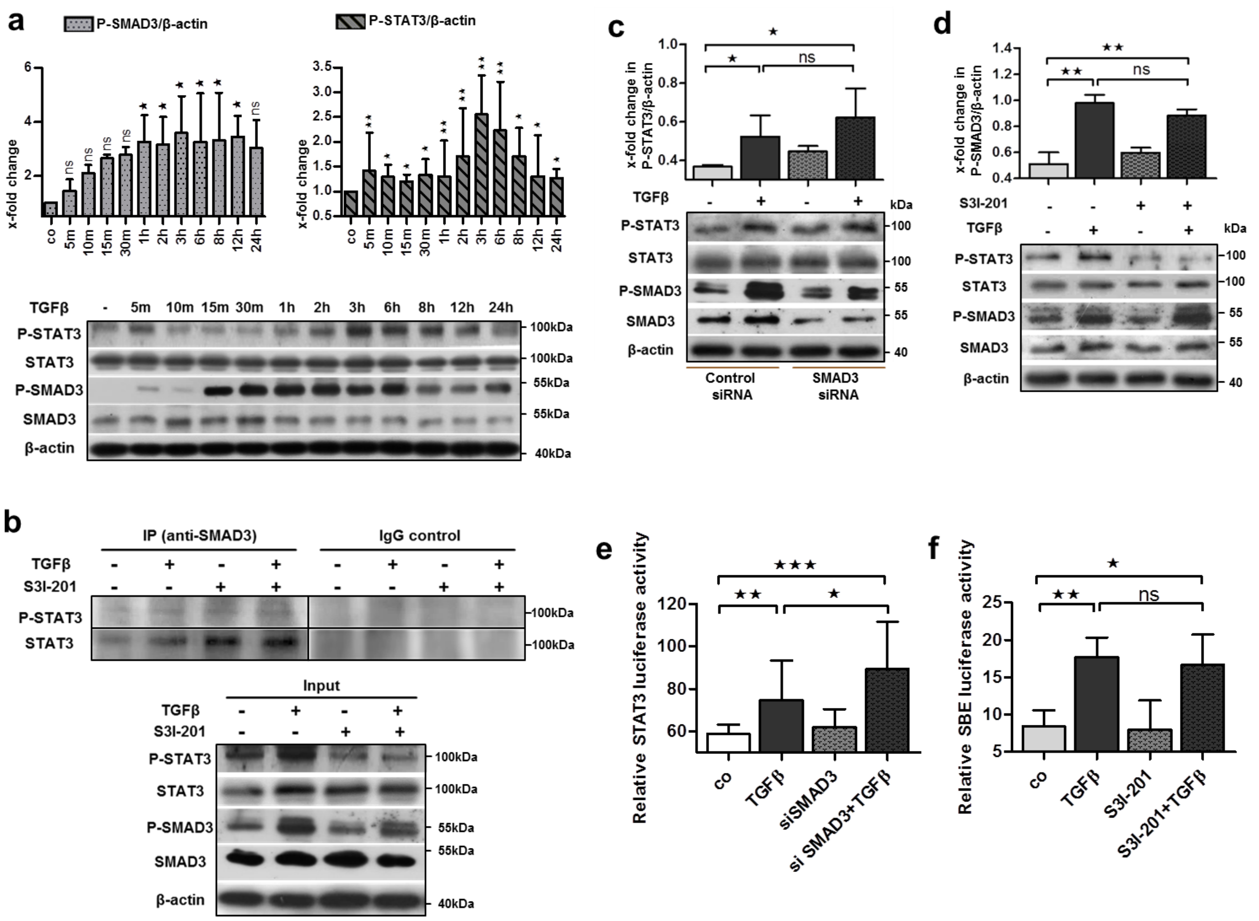

Supplementary Figure 3: Interaction of STAT3 and SMAD3 signaling in human fibroblasts stimulated with TGFβ

(a) Representative Western blots and quantification of P-STAT3 and P-SMAD3 in cultured human dermal fibroblasts stimulated with TGFβ for different time periods. **(b)** Representative images of SMAD3 co-immunoprecipitates of fibroblasts with and without TGFβ and S3I-201. **(c;e)** Effects of siRNA-mediated knockdown of SMAD3 on TGFβ-induced STAT3 phosphorylation in human fibroblasts determined by **(c)** Western blot analyses and **(e)** reporter activity in fibroblasts transfected with STAT3 luciferase reporter plasmid. **(d;f)** Effects of pharmacological inhibition of STAT3 on TGFβ-induced SMAD3 phosphorylation in human fibroblasts determined by **(d)** Western blot analyses and **(f)** reporter activity in fibroblasts transfected with SBE luciferase reporter plasmid. Expected band size for P-STAT3 and STAT3 are 79 kDa (lower faint band) and 86 kDa (higher intense band) and the ladder represents 100kDa. Beta-actin expected molecular weight/size is 42 kDa. The ladder for SMAD3 and P-SMAD3 represents 55 kDa asnd the expected band size is 50kDa. Luciferase activity was normalized against internal non-inducible Renilla luciferase activity. n ≥ 4 cell-lines with 2 technical replicates for all experiments. Results are shown as median ± interquartile range (IQR). Significance was determined by Mann-Whitney test. *p<0.05; **p<0.01, ***p<0.001 versus healthy dermal fibroblasts or TGFβ-stimulated fibroblasts, respectively.

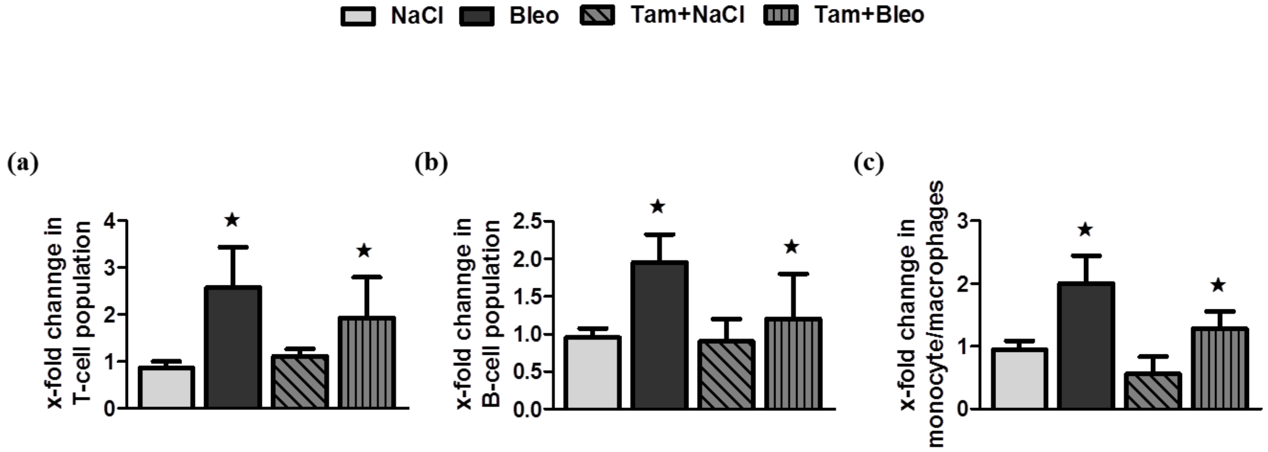

Supplementary Figure 4: Changes in the numbers of inflammatory leukocytes in bleomycin-induced skin fibrosis.

The total number of cell counts with specific cell-type marker using microscopic analysis in the skin tissue sections from the experimental mouse model of bleomycin-induced skin fibrosis in mice with fibroblast-specific, tamoxifen-inducible knockout of STAT3 in STAT3 and control littermates (C57Bl/6 background, 12 weeks of age). n ≥ 6 mice per group. (a) CD3 positive cells / T cells. (b) B220-positive cells / B cells. (c) CD11b-positive cells / monocytes and macrophages. Results are shown as median ± interquartile range (IQR). Significance was determined by Mann-Whitney test, as compared to vehicle-treated non-fibrotic control mice. *$p<0.05$. Tam: Tamoxifen, Bleo: Bleomycin.

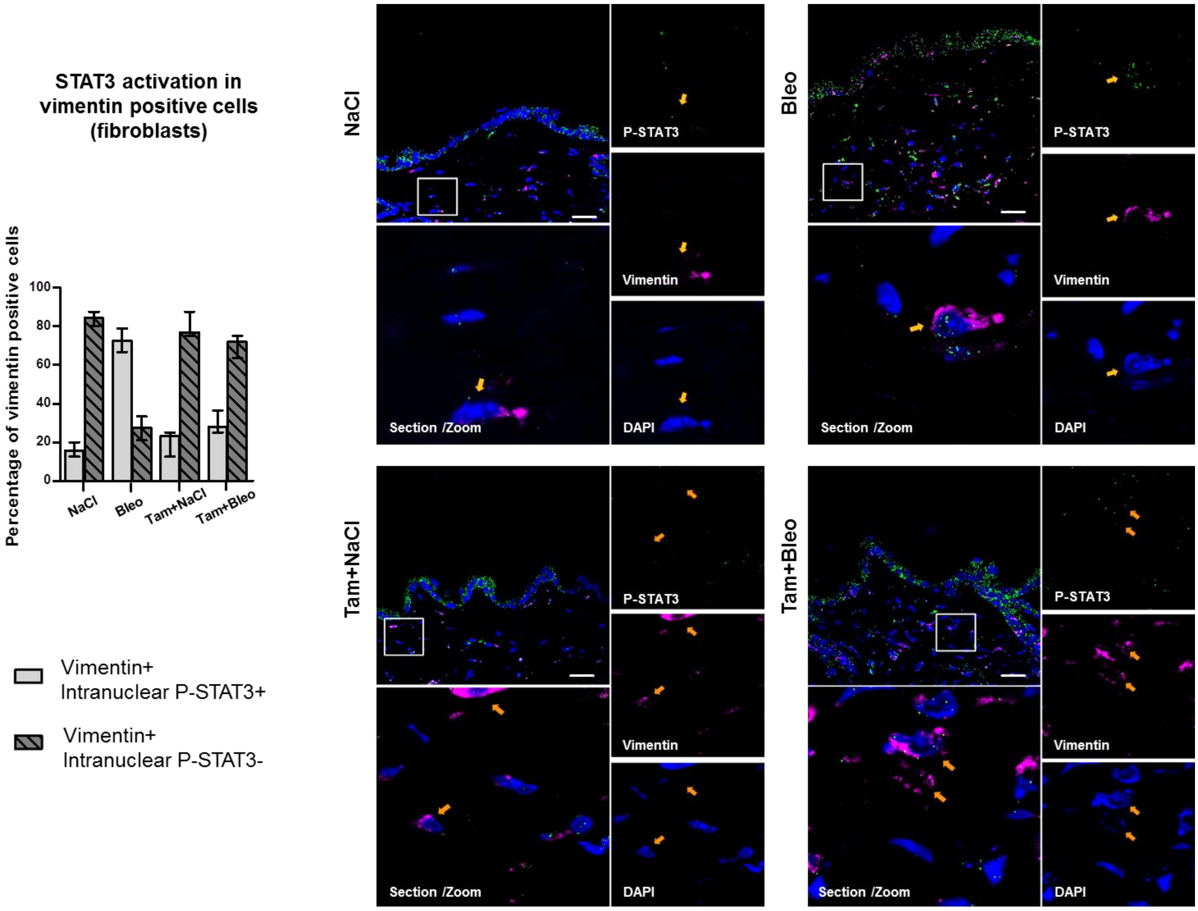

Supplementary Figure 5: Expression of P-STAT3 in fibroblasts upon fibroblast-specific knockout of STAT3 in bleomycin-induced fibrosis.

Confocal microscopy analysis of immunofluorescent stainings of P-STAT3 (green) co-stained with fibroblast-specific marker vimentin (magenta) and DAPI (staining of nuclei) in skin tissue sections from the experimental mouse model of bleomycin-induced skin fibrosis in mice with fibroblast-specific, tamoxifen-inducible, Cre-loxP–based (Col1a2-Cre-ER) knockout of STAT3 in STAT3$^{fl/fl}$ mice and control littermates (C57Bl/6background, 12 weeks of age). n ≥ 6 mice per group. Representative confocal images are shown with a horizontal scale bar of 50 µm and a zoom factor of 6.4 (right). Quantification of the percentage of cells expressing vimentin and nuclear P-STAT3 per total cells expressing vimentin (as indicated by arrows in the representative images) is shown. The number of P-STAT3 positive fluorescent cells was counted in ≥ 6 high-power fields per mouse. Tam: Tamoxifen; Bleo: Bleomycin.

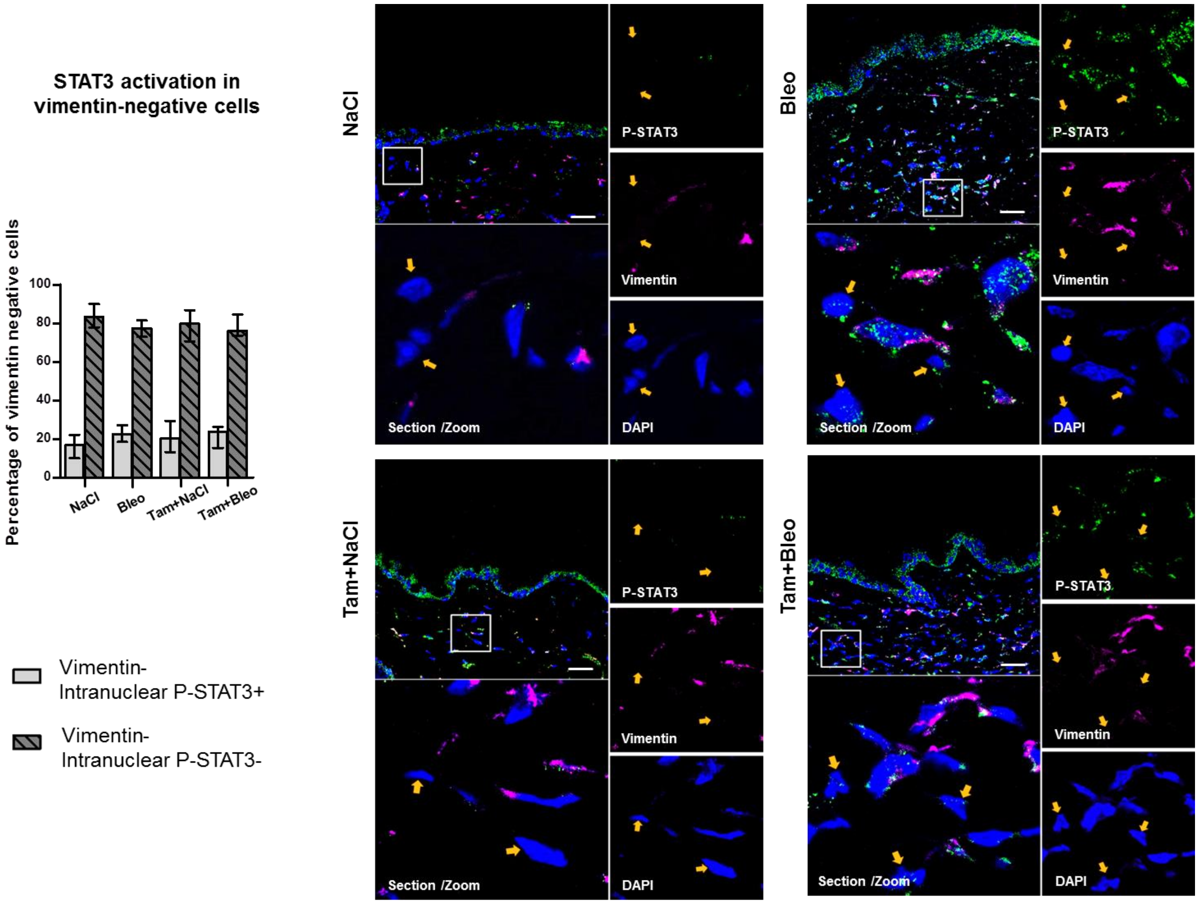

Supplementary Figure 6: Expression of P-STAT3 in vimentin-negative cells upon fibroblast-specific knockout of STAT3 in bleomycin-induced fibrosis.

Confocal microscopy analysis of immunofluorescent stainings of P-STAT3 (green) co-stained with fibroblast- specific marker vimentin (magenta) and DAPI (staining of nuclei) in skin tissue sections from the experimental mouse model of bleomycin-induced skin fibrosis in mice with fibroblast-specific, tamoxifen-inducible, Cre-loxP–based (Col1a2-Cre-ER) knockout of STAT3 in STAT3$^{fl/fl}$ mice and control littermates (C57Bl/6background, 12 weeks of age). n ≥ 6 mice per group. Representative confocal images are shown with a horizontal scale bar of 50 µm and a zoom factor of 6.4 (right). Quantification of the percentage of cells not stained with vimentin and nuclear P-STAT3 per total cells not expressing vimentin (as indicated by arrows in the representative images) is shown. The number of P-STAT3 positive fluorescent cells was counted in ≥ 6 high-power fields per mouse. Tam: Tamoxifen; Bleo: Bleomycin.

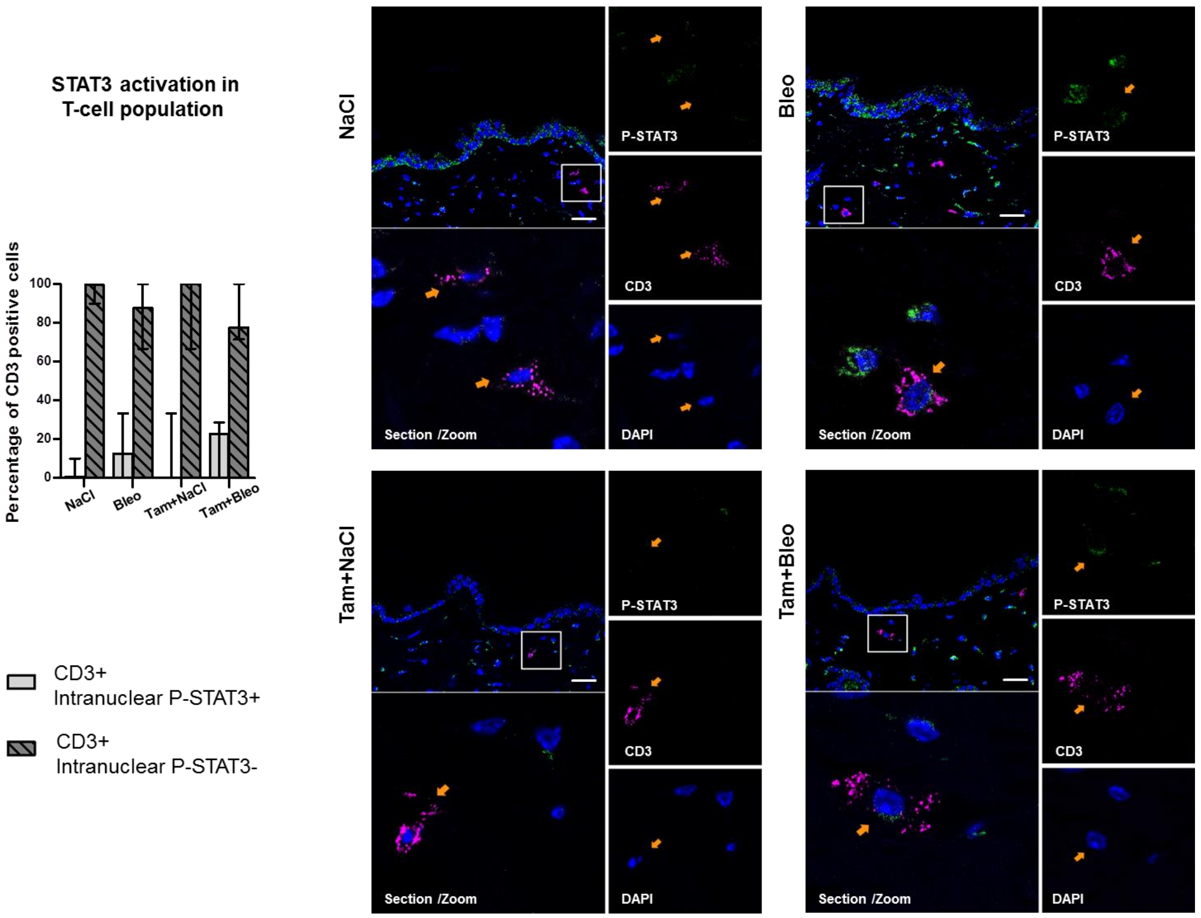

Supplementary Figure 7: Expression of P-STAT3 in CD3 positive cells / T cells upon fibroblast-specific knockout of STAT3 in bleomycin-induced fibrosis.

Confocal microscopy analysis of immunofluorescent stainings of P-STAT3 (green) co-stained with T cell-specific marker CD3 (magenta) and DAPI (staining of nuclei) in skin tissue sections from the experimental mouse model of bleomycin-induced skin fibrosis in mice with fibroblast-specific, tamoxifen-inducible, Cre-loxP–based (Col1a2-Cre-ER) knockout of STAT3 in STAT3$^{fl/fl}$ mice and control littermates (C57Bl/6background, 12 weeks of age). n ≥ 6 mice per group. Representative confocal images are shown with a horizontal scale bar of 50 µm and a zoom factor of 6.4 (right). Quantification of the percentage of cells expressing CD3 and nuclear P-STAT3 per total cells expressing T-cell marker CD3 (as indicated by arrows in the representative images) is shown. The number of P-STAT3 positive fluorescent cells was counted in ≥ 6 high-power fields per mouse. Tam: Tamoxifen; Bleo: Bleomycin.

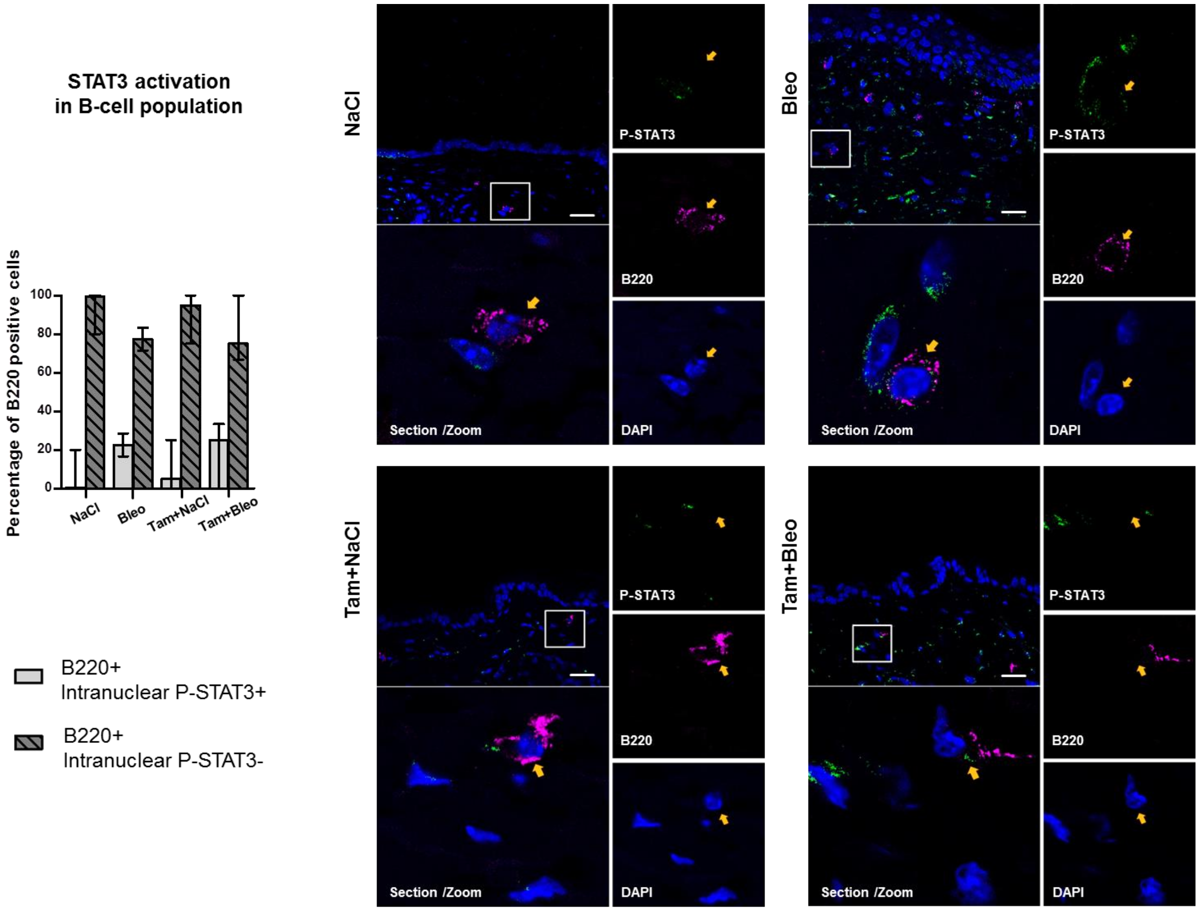

Supplementary Figure 8: Expression of P-STAT3 in B220-positive cells / B cells upon fibroblast-specific knockout of STAT3 in bleomycin-induced fibrosis.

Confocal microscopy analysis of immunofluorescent stainings of P-STAT3 (green) co-stained with B-cell specific marker B220 (magenta) and DAPI (staining of nuclei) in skin tissue sections from the experimental mouse model of bleomycin-induced skin fibrosis in mice with fibroblast-specific, tamoxifen-inducible, Cre-loxP–based (Col1a2-Cre-ER) knockout of STAT3 in STAT3$^{fl/fl}$ mice and control littermates (C57Bl/6background, 12 weeks of age). n ≥ 6 mice per group. Representative confocal images are shown with a horizontal scale bar of 50 µm and a zoom factor of 6.4 (right). Quantification of the percentage of cells with the B-cell marker B220 and nuclear P-STAT3 per total cells expressing B220 (as indicated by arrows in the representative images) is shown. The number of P-STAT3 positive fluorescent cells was counted in ≥ 6 high-power fields per mouse. Tam: Tamoxifen; Bleo: Bleomycin.

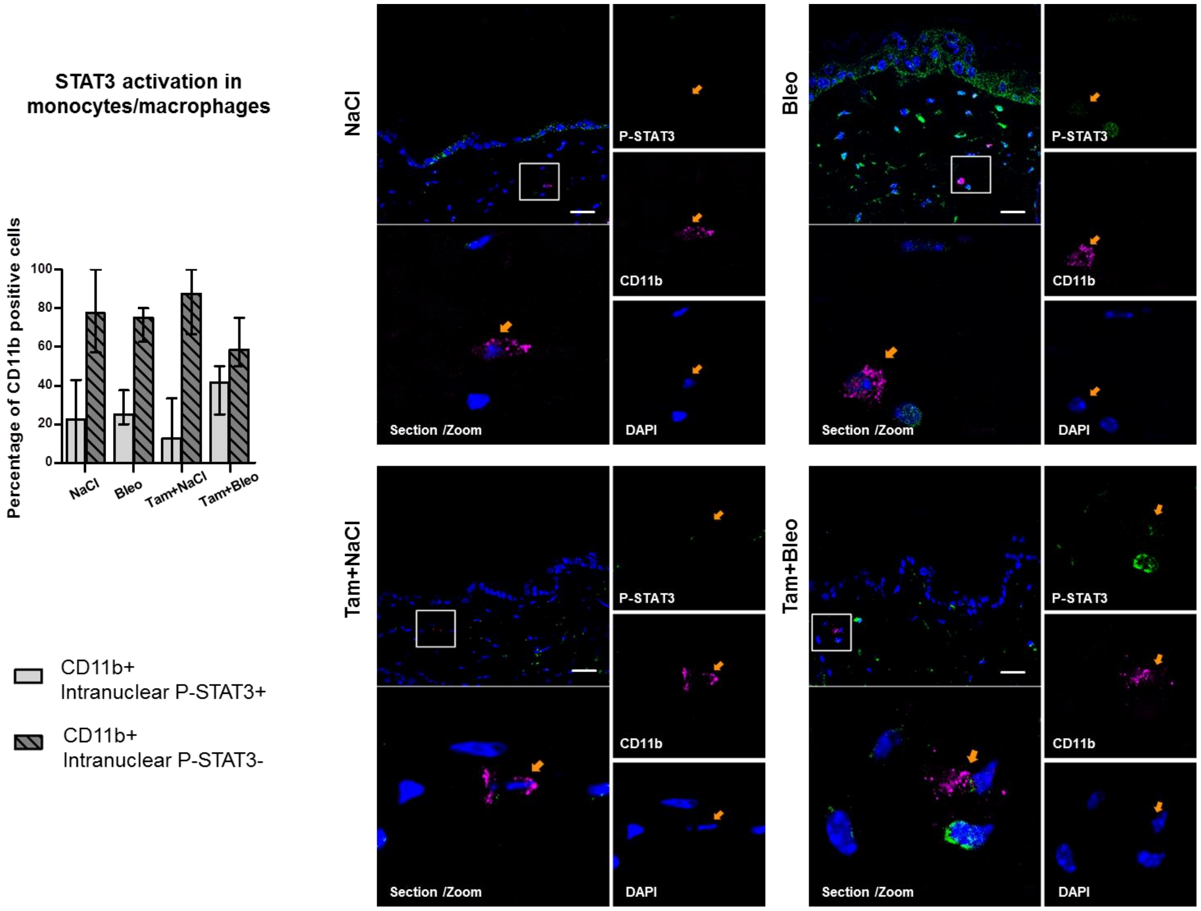

Supplementary Figure 9: Expression of P-STAT3 in CD11b-positive cells / monocytes and macrophages upon fibroblast-specific knockout of STAT3 in bleomycin-induced fibrosis.

Confocal microscopy analysis of immunofluorescent stainings of P-STAT3 (green) co-stained with monoycte and macrophage marker CD11b (magenta) and DAPI (staining of nuclei) in skin tissue sections from the experimental mouse model of bleomycin-induced skin fibrosis in mice with fibroblast-specific, tamoxifen-inducible, Cre-loxP–based (Col1a2-Cre-ER) knockout of STAT3 in STAT3$^{fl/fl}$ mice and control littermates (C57Bl/6background, 12 weeks of age). n ≥ 6 mice per group. Representative confocal images are shown with a horizontal scale bar of 50 µm and a zoom factor of 6.4 (right). Quantification of the percentage of cells with monocytes and macrophage marker CD11b and nuclear P-STAT3 per total cells expressing CD11b (as indicated by arrows in the representative images) is shown. The number of P-STAT3 positive fluorescent cells was counted in ≥ 6 high-power fields per mouse. Tam: Tamoxifen; Bleo: Bleomycin.

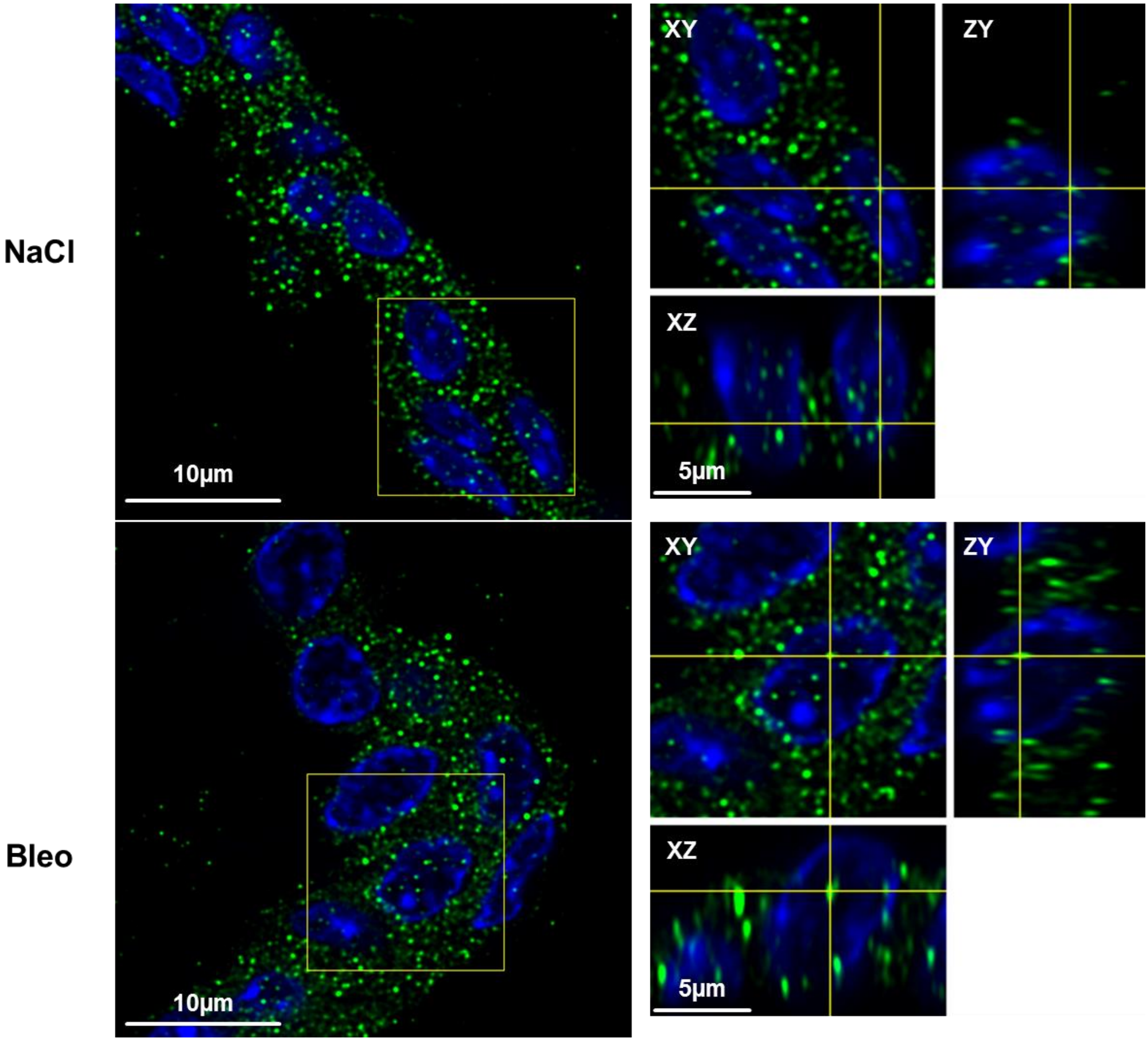

Supplementary Figure 10: Challenge with bleomycin does not alter the levels of P-STAT3 in epidermal keratinocytes.

Representative confocal images of immunofluorescent stainings of P-STAT3 (green) co-stained with nuclear marker DAPI (blue) in skin tissue sections from the experimental mouse model of bleomycin-induced skin fibrosis (C57Bl/6background, 12 weeks of age). Mice injected with NaCl served as controls. n ≥ 4 mice with 4 technical replicates per group. Quantitative immunofluorescence analysis revealed similar ratio of nuclear P-STAT3 / total staining in mice challenged with bleomycin to that of the non-fibrotic controls.

Additionally, videos containing a series of z-stacks captured throughout the depth of a random area of epidermis of skin tissue of bleomycin-challenged mice along with that of the non-fibrotic control are available with the uploaded material.

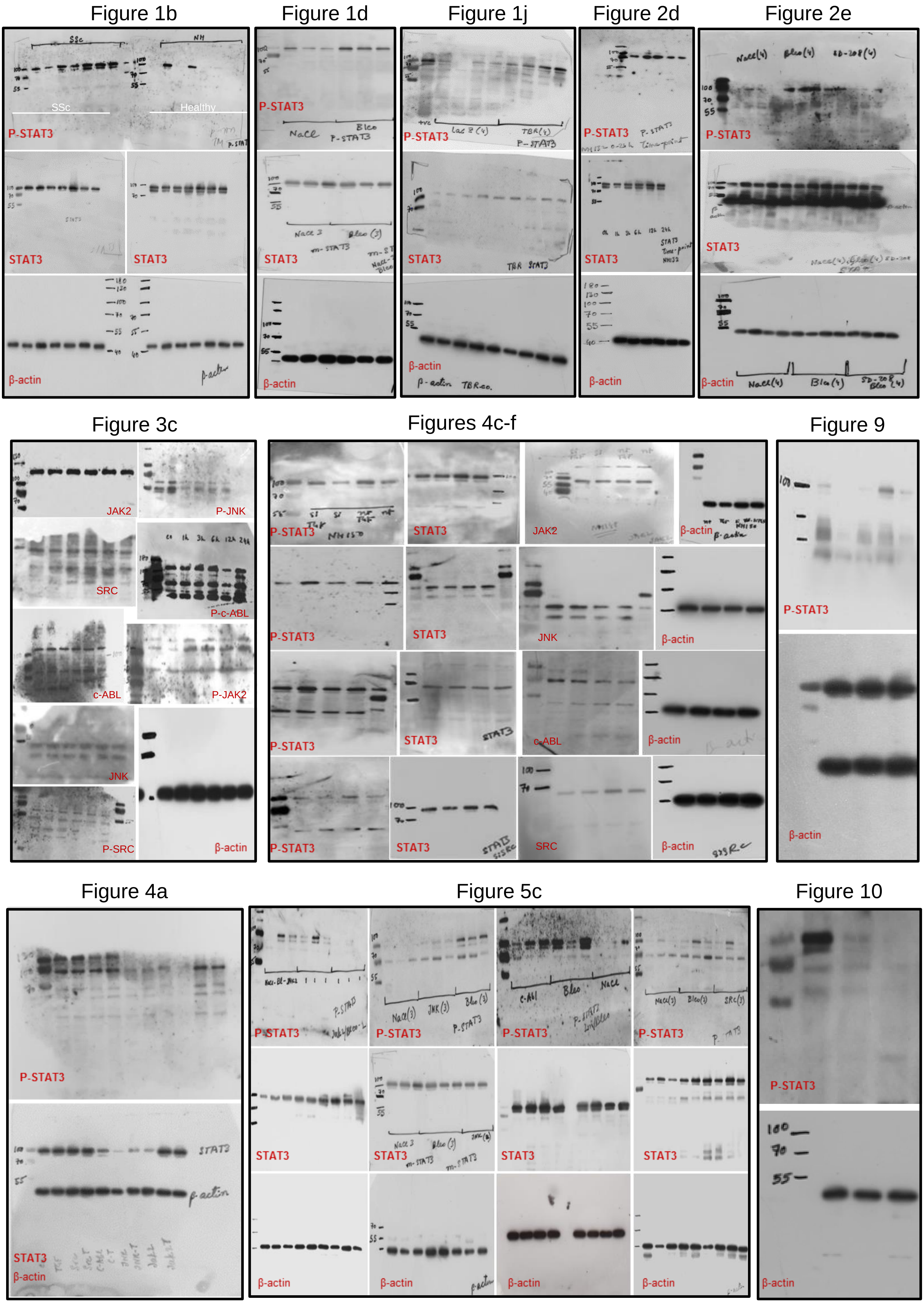

Supplementary Figure 11: Original uncropped scans of Western blots

Supplementary Table 1: Clinical characteristic of patients with systemic sclerosis (SSc)

Characteristics	
Gender (F/M)	15 /12
Age (years), median (range)	47 (18 – 67)
Disease duration (years), median (range)	4 (1 – 11)
Disease subset (limited vs. diffuse cutaneous)	13/ 14
ANA positive	20
Anti-topoisomerase-autoantibodies	7
Anti-centromere-autoantibodies	8
Pulmonary fibrosis	14
Myocardial involvement	6
Pulmonary arterial hypertension	5
Digital ulcers	13
Active disease	9
Medication	No DMARDs, Corticosteroids or NSAIDs at the time of biopsy

Supplementary Table 2: siRNA sequences for gene silencing

Gene	Sequences
JAK1	5′-GCGAUAUAUUCCAGAAACAdTdT-3′
JAK2	5′-TTTGGCAACAGACAAATGGAdTdT-3′
c-ABL	5′-GUUGGUUCAUCAUCAUUCAdTdT-3′
SRC	5′-GACAGAGCCAGGAUUUGAAdTdT-3′
SMAD3	5′- GGAGAAAUGGUGCGAGAAGdTdT -3′

Supplementary Table 3: Real-time qRT-PCR primers

Gene	Primer sequences
ACTA2	Forward primer: 5’-AAGAGGAATCCTGACCCTGAA-3’<br>Reverse primer: 5’-TGGTGATGATGCCATGTTCT-3’
ACTB	Forward primer: 5’-GACACCTCGCGGGCTCTGC-3’<br>Reverse primer: 5’-CGCCAGGCCTCCTGGAAACG-3’
COL1A1	Forward primer: 5’-GGTCAGCACCACCGATGTC-3’<br>Reverse primer: 5’-ATGGTACCTGAGGCCGTTC-3’
COL1A2	Forward primer: 5’-GGTCAGCACCACCGATGTC-3’<br>Reverse primer: 5’-CACGCCTGCCCTTCCTTT-3’
CTGF	Forward primer: 5’-AACTCACACAACAACTCTTCCCCGC-3’<br>Reverse primer: 5’-GAGTCGCACTGGCTGTCTCCTCT-3’
Acta2	Forward primer: 5’-ATGCCTCTGGACGTACAACTG-3’<br>Reverse primer: 5’-CACACCATCTCCAGAGTCCA-3’
Actb	Forward primer: 5’-GACACCTCGCGGGCTCTGC-3’<br>Reverse primer: 5’-GTCCACGGGCCTGTCTCGC-3’
Col1a1	Forward primer: 5’-GAAGCACGTCTGGTTTGGA-3’<br>Reverse primer: 5’-ACTCGAACGGGAATCCATC-3’
Col1a2	Forward primer: 5’-TCAAACTGGCTGCCACCAT-3’<br>Reverse primer: 5’-CCAACAAGCATGTCTGGTTAGGA-3’
Comp	Forward primer: 5’-CCAGACCAGAGGGATGTGGA-3’<br>Reverse primer: 5’-CTGGGCACTGTTGGGTACTG-3’
Ctgf	Forward primer: 5’-CTGCCTACCGACTGGAAGAC-3’<br>Reverse primer: 5’-TCGCATCATAGTTGGGTCTG-3’
Pai-1	Forward primer: 5’-ACGTTGTGGAACTGCCCTAC-3’<br>Reverse primer: 5’-AGCGATGAACATGCTGAGG-3’
Thbs-1	Forward primer: 5’-TAGCTGGAAATGTGGTGCGT-3’<br>Reverse primer: 5’-GGCACTTCTTTGCACTCATCG-3’
