# Supplementary material for: Activation of STAT3 integrates common profibrotic pathways to promote fibroblast activation and tissue fibrosis
Source: Nat Commun. 2017 Oct 24;8:1130. doi: 10.1038/s41467-017-01236-6 (PMC5654983; doi:10.1038/s41467-017-01236-6)
Supplement: Supplementary file 2 — Description of Additional Supplementary Files [file 41467_2017_1236_MOESM2_ESM.pdf]

Description of Supplementary Files

File name: Supplementary Movie 1
Description: A series of z-stacks captured throughout the depth of a random area of epidermis of skin tissue of non-fibrotic mice injected with NaCl.

File name: Supplementary Movie 2
Description: A series of z-stacks captured throughout the depth of a random area of epidermis of skin tissue of bleomycin-challenged mice.
